# Restriction of growth and biofilm formation of *ESKAPE* pathogens by caprine gut-derived probiotic bacteria

**DOI:** 10.3389/fmicb.2024.1428808

**Published:** 2024-07-29

**Authors:** Prerna Saini, Repally Ayyanna, Rishi Kumar, Sayan Kumar Bhowmick, Vinay Bhaskar, Bappaditya Dey

**Affiliations:** ^1^National Institute of Animal Biotechnology, Hyderabad, India; ^2^Regional Centre for Biotechnology, Faridabad, India

**Keywords:** probiotics, *Lactobacillus*, *ESKAPE* pathogens, biofilm, antimicrobial resistance

## Abstract

The accelerated rise in antimicrobial resistance (AMR) poses a significant global health risk, necessitating the exploration of alternative strategies to combat pathogenic infections. Biofilm-related infections that are unresponsive to standard antibiotics often require the use of higher-order antimicrobials with toxic side effects and the potential to disrupt the microbiome. Probiotic therapy, with its diverse benefits and inherent safety, is emerging as a promising approach to prevent and treat various infections, and as an alternative to antibiotic therapy. In this study, we isolated novel probiotic bacteria from the gut of domestic goats (*Capra hircus*) and evaluated their antimicrobial and anti-biofilm activities against the ‘*ESKAPE*’ group of pathogens. We performed comprehensive microbiological, biochemical, and molecular characterizations, including analysis of the 16S-rRNA gene V1-V3 region and the 16S-23S ISR region, on 20 caprine gut-derived lactic acid bacteria (LAB). Among these, six selected *Lactobacillus* isolates demonstrated substantial biofilm formation under anaerobic conditions and exhibited robust cell surface hydrophobicity and autoaggregation, and epithelial cell adhesion properties highlighting their superior enteric colonization capability. Notably, these *Lactobacillus* isolates exhibited broad-spectrum growth inhibitory and anti-biofilm properties against ‘*ESKAPE*’ pathogens. Additionally, the *Lactobacillus* isolates were susceptible to antibiotics listed by the European Food Safety Authority (EFSA) within the prescribed Minimum Inhibitory Concentration limits, suggesting their safety as feed additives. The remarkable probiotic characteristics exhibited by the caprine gut-derived *Lactobacillus* isolates in this study strongly endorse their potential as compelling alternatives to antibiotics and direct-fed microbial (DFM) feed supplements in the livestock industry, addressing the escalating need for antibiotic-free animal products.

## Introduction

Antimicrobial resistance (AMR) is a growing global public health concern because many pathogens are becoming resistant to standard antibiotics. The ‘*ESKAPE*’ group of six nosocomial pathogens is leading the priority pathogen list of multidrug-resistant (MDR) and extensively drug-resistant (XDR) bacteria that includes, i.e., *Enterococcus faecium, Staphylococcus aureus, Klebsiella pneumoniae, Acinetobacter baumannii, Pseudomonas aeruginosa, and Enterobacter* spp. (Boucher et al., 2009). These pathogens can escape the bactericidal actions of various antimicrobial agents. Inappropriate use or overuse of antibiotics has resulted in the global emergence and spread of these pathogens, causing outbreaks, community-acquired infections, and transmission Infection-related fatalities caused by drug-resistant (DR) pathogens are expected to account for the largest number of deaths worldwide by 2050 (Shankar, 2016). Biofilm formation is a major mechanism by which DR and MDR-*ESKAPE* bacteria exhibit a drug resistance phenotype (Mulani et al., 2019). Biofilms protect specialized dormant persister cells that are tolerant to antibiotics, as well as host immune cells, leading to difficult-to-treat recalcitrant infections (Lewis, 2005). Antibiotics are administered alone or in combination to effectively treat these infections. However, with every passing year, the number of antibiotics to treat these infections is declining, predisposing humanity towards a future with fewer antibiotics that will probably become ineffective in the near future (Andersson et al., 2020). Hence, there is a dire need to find safe and natural alternative antibiotic agents, such as probiotics, to treat infections caused by such pathogens (Mulani et al., 2019; da Rosa et al., 2020).

Probiotics are live microorganisms that confer health benefits to the host when administered in adequate amounts and are considered a potential alternative to antibiotics (Hill et al., 2014). Most probiotics belong to lactic acid bacteria (LAB), a group of bacteria that are generally regarded as safe (GRAS), and are the oldest known probiotic to inhibit or treat infections caused by DR pathogens (Mattia and Merker, 2008; Silva et al., 2020). LAB strains belonging to *Lactobacillus* and *Bifidobacterium* genera are known to inhibit pathogens by a plethora of mechanisms, including competitive exclusion, adhesion to the intestinal mucosa, host immunomodulation, enhancement of epithelial barrier integrity, and production of organic acids, hydrogen peroxide, bacteriocins, and antimicrobial peptides etc. (Bermudez-Brito et al., 2012). The application of *Lactobacillus* has shown promise for treating infections caused by *ESKAPE* bacteria in both animals and humans. For example, topical application of *Lactobacillus acidophilus (L. acidophilus) or Limosilactobacillus reuteri (Lm. reuteri)* was effective in treating wound infections caused by *A. baumanii* (Stanbro et al., 2020; Todorov et al., 2023). The application of *Limosilactobacillus fermentum (Lm. fermentum)* improved the condition of ischemic wounds in rabbits (Jones et al., 2012). Similar activities against skin pathogens, such as *E. coli, P. aeruginosa, S. aureus,* and *Propionibacterium* have been reported for *Lactiplantibacillus plantarum (Lp. plantarum) ATCC 10241* and *Lactobacillus delbrueckii (Lb. delbrueckii) DSMZ 20081* (Fijan et al., 2019; Yilmaz and Turkyilmaz, 2023). Addition*ally, Lacticaseibacillus rhamnosus (Ls. rhamnosus), Lm. fermentum, L. acidophilus, and Lp. plantarum* prevented the adhesion and regrowth of *E. faecalis and E. faecium* biofilms (Velraeds et al., 1996). *Lactobacillus gasseri (L. gasseri)* LBM220 isolated from the feces of breastfed infants showed strong antibacterial activity against all six MDR-*ESKAPE* pathogens (Rastogi et al., 2021). *Lactobacillus* can inhibit the growth of a variety of livestock pathogens, including bovine mastitis-causing *Methicillin Resistant Staphylococcus aureus* (MRSA), which also causes skin abscesses and septicemia (Fitzgerald, 2012; Kang et al., 2017). Gram-negative bacillus *K. pneumoniae*, a common causative agent of clinical mastitis in dairy cattle, is an emerging zoonotic and foodborne pathogen worldwide (Munoz et al., 2006; Darniati et al., 2021). *Lp. plantarum CIRM653* impaired *K. pneumoniae* biofilms independently of its bactericidal effect (Lagrafeuille et al., 2018). *Lb. delbrueckii subsp. delbrueckii* LDD01 also showed the highest inhibitory effect against *K. pneumoniae* (Mogna et al., 2016). *Pseudomonas aeruginosa* is also associated with many diseases in livestock and companion animals, including urinary tract infections in dogs, mastitis in dairy cows, and endometritis in horses (Leitner and Krifucks, 2007; Haenni et al., 2015). Growth inhibition and anti-biofilm effects of *L. fermentum* against MDR, XDR, and pan-drug-resistant (PDR) *P. aeruginosa* strains was also reported (Shokri et al., 2018). Notably, the *L. acidophilus* ATCC 4356 strain is well known for its strong inhibition of biofilm growth against a majority of *P. aeruginosa* strains (Alexandre et al., 2014; Elbadri et al., 2019). European Food Safety Authority (EFSA) has listed *E. faecalis* as an opportunistic pathogen in birds, poultry, and reptiles [EFSA Panel on Animal Health and Welfare (AHAW) et al., 2022]. *Enterococcus faecalis* has also been detected in animals, meat, and meat-based products, as well as in human fecal samples and in patients with bloodstream infections (Hammerum, 2012). Biosurfactants and conditioned media from several probiotic bacteria were found to prevent adhesion and biofilm formation by *E. faecalis* (Safadi et al., 2022). Lipoteichoic acids from *Lp. plantarum* have been proven effective in disrupting mature *E. faecalis* biofilms (Kim et al., 2020). Furthermore, livestock animals are the main reservoir of Shiga toxin-producing *E. coli* with zoonotic potential, and several *Lactobacillus* isolates exhibit antagonistic activity against these *E. coli* strains (Frank and Marth, 1977; Byakika et al., 2019).

Since the antibacterial properties of probiotic *Lactobacillus* depend on strain specificity, comprehensive characterization and evaluation of the probiotic properties of new or novel *Lactobacillus* isolates from natural sources are essential before selecting them for preclinical evaluation for their potential use (Ramos et al., 2013; Campana et al., 2017). To date, no study has demonstrated the isolation and inhibitory effects of Caprine gut-derived *Lactobacillus* against *ESKAPE* group pathogens. Here, we isolated *Lactobacillus* from the small intestine of domestic goats, identified them by biochemical and molecular methods, and assessed their probiotic properties, such as acid and bile tolerance, surface properties, epithelial cell adhesion, intrinsic colonization-*cum*-biofilm formation ability, and antibiotics susceptibility, and finally demonstrated their antagonistic and anti-biofilm effects against *ESKAPE* group pathogens.

## Materials and methods

### Bacterial strains and growth conditions

The *Lp. plantarum* strain (MTCC-2621) used as the positive control strain was procured from the Microbial Type Culture Collection (MTCC), CSIR-Institute of Microbial Technology (IMTECH), Chandigarh, India. *Lactobacillus* isolates were grown in MRS agar or broth, as applicable. Pathogenic *ESKAPE* group bacteria were obtained from: *E. coli* and *S. aureus* (American Type Culture collection-ATCC, United States), *K. pneumonae*, *A. baumanii*, *P. aeruginosa* (MTCC, Chandigarh, India), and *E. faecalis* (National Center for Microbial Resources-NCMR, Pune, India). All bacteria were subcultured from glycerol stocks in Luria Bertani (LB) broth at 37°C overnight in a shaker incubator. Frozen glycerol stocks were prepared and stored at −80°C for future use.

### Isolation of lactic acid bacteria from goat intestinal tissue

Intestinal tissues (jejunum) were collected from goats (n = 11) immediately after slaughter in the Govt. authorized slaughterhouse in chilled phosphate-buffered saline (PBS, pH 7.4), transported on ice to the laboratory, and processed on the same day for bacterial isolation in a BSL-2 facility. Goat intestinal tissue samples were thoroughly washed with 1XPBS to remove intestinal content and debris. Each intestinal tissue sample was dissected into small pieces and collected in screw-capped 2 mL homogenization tubes containing 1.0 mm glass beads. Tissue sections were homogenized twice for 30 s at 4,000 rpm using a bead beater (BeadBug™). Subsequently, 100 μL of the homogenate was inoculated into 10 mL of sterile MRS broth. In parallel, the intestinal content was collected separately in a 1.5 mL tube, and 100 μL of the soup was directly inoculated into 10 mL of MRS broth. All the samples were incubated at 37°C in an orbital shaker incubator at 200 rpm for 24 h. Subsequently, cultures were 10-fold serially diluted up to 1: 10^6^, and 100 μL samples from the three highest dilutions were plated onto MRS agar plates and incubated at 37°C aerobically for 48 h. Single isolated colonies were picked and replated onto MRS agar for colony morphology evaluation and subsequent characterization. Typically, round, creamy-white, shiny colonies with smooth and proper margins were selected based on the morphology description in the Bergey’s Manual of Determinative Bacteriology.

### Biochemical characterization

Each colony was screened for colony and bacterial morphology, Gram staining, and catalase activity. Only Gram-positive and catalase-negative rods were characterized further. Various other tests were performed to characterize *Lactobacillus*, as recommended in Bergey’s Manual of Determinative Bacteriology, including the indole test for the production of indole from tryptophan, the nitrate test for the reduction of nitrate to nitrite, and the Vogues Proskauer (VP) test to determine the presence of acetyl methyl carbinol after glucose fermentation (Bergey, 1994). The carbohydrate fermentation ability of all isolates was assessed using the Himedia Hilacto identification kit (KB020, Himedia), which is a standardized colorimetric identification system based on pH change and substrate utilization for the genus *Lactobacillus*. The kit contained one strip that included 12 wells, one for esculin and another for catalase, and 10 wells for 10 different carbohydrate sugars, that is, xylose, cellobiose, arabinose, maltose, galactose, mannose, melibiose, raffinose, sucrose, and trehalose. 50 μL of 0.1 OD (A_600nm_) bacterial inoculum (50 μL) was added to each well of a strip by surface inoculation and incubated at 37°C for 24–48 h. For carbohydrate fermentation and esculin tests, a color change was observed in each well, according to the manufacturer’s protocol. For the catalase test, a loopful of growth was scraped from the surface of the plate and dipped in a clean glass test tube containing 3% of freshly prepared H_2_O_2_, and effervescence was observed on the surface of the loop. No effervescence was observed in the case of a negative catalase test.

### Molecular identification of *Lactobacillus* isolates

To identify the genus of the *Lactobacillus* isolates, colony-PCR was carried out on the genomic DNA extracted from the *Lactobacillus* isolates through the Triton-X-100 boiling lysis method using previously reported *Lactobacillus* genus-specific primers Forward-R16-1 and Reverse-LbLMA1 as described previously (Supplementary Table S1) (Dubernet et al., 2002). The optimized PCR cycling conditions included initial denaturation at 94°C for 1 min, followed by 30 cycles consisting of denaturation at 98°C for 5 s, annealing at 55°C for 5 s, and extension at 72°C for 5 s, and 2 min of final extension. The amplified PCR products were analyzed using agarose gel electrophoresis with ethidium bromide staining and visualized under UV light.

For *Lactobacillus* species identification, a second round of PCR was performed, targeting the 16S-rRNA V1-V3 region and 16S-23S Intergenic Spacer Region (ISR). Two separate primer pair sets were used to amplify ~509 bp and ~ 565 bp, using 16S(8-27)-F and V3(519-536)-R, and 16S(ISR)-F and 23S(ISR)-R primer pairs, respectively (Supplementary Table S1) (Weisburg et al., 1991; Gurtler and Stanisich, 1996; Turner et al., 1999). The first PCR was conducted using sequencing primer set-1 under the following conditions: initial denaturation at 95°C for 10 min, followed by 40 cycles of denaturation at 95°C for 30 s, annealing at 57°C for 30 s, extension at 72°C for 60 s, and final extension of 7 min. For primer set-2, PCR conditions involved an initial denaturation at 95°C for 10 min, followed by 40 cycles of denaturation at 95°C for 30 s, annealing at 59°C for 30 s, extension at 72°C for 60 s, and a final extension of 7 min. Amplified PCR products were subjected to agarose gel electrophoresis and ethidium bromide staining. PCR products were subsequently excised from the agarose gel and DNA was extracted using the Qiagen Gel Extraction Kit (#28704) according to the manufacturer’s protocol. The excised amplicons were sequenced by Sanger sequencing, using the forward primers for each PCR described above. The trimmed sequences were subjected to NCBI BLAST analysis using default parameters to identify the closest bacterial species via % sequence cover and percentage identity.

Subsequently, phylogenetic analysis of the 12 *Lactobacillus* isolates was performed using either the sequences of the 16S rRNA-V1-V3 region or 16S-23S ISR via hierarchical clustering using the neighbor-joining method. Briefly, both the sequences were aligned using ClustalW (default parameters). The alignment results were then used to determine the best-fit nucleotide-substitutional model. The Kimura 2-parameter and Tamura 3-parameter models were used for the V3 and ISR regions, respectively. The neighbor-joining method (bootstrap test −1,000 replicates) was used to generate the phylogenetic trees. Evolutionary analyses were conducted using MEGA11 software.

### Acid and bile tolerance test

The pH and acid tolerance of the samples were assessed under three different conditions (pH 2.0, 3.0, and 4.0) by adjusting the pH of MRS broth. In brief, 96-well microplates were added with 150 μL/well of MRS broth, adjusted to three different pH levels, and inoculated with 1% (v/v) bacterial culture from an overnight broth culture, which had been adjusted to 0.8 OD (A_600nm_). The microplates were incubated at 37°C for 12 h at 200 rpm in a microplate incubator. Absorbance was read at 600 nm at two-hour intervals for a total of 12 h.

To evaluate the resistance of *Lactobacillus* isolates to high bile salt conditions, the isolates were cultured in MRS broth containing three different bile salt concentrations: 0.5, 1, and 2% w/v. The same culture method was employed as described above for pH shock, with the exception that standard MRS medium (pH 5.5) was used as the base medium along with the appropriate bile salt concentration.

### Cell surface hydrophobicity assay

The cell surface hydrophobicity of Lactobacillus isolates was determined using the microbial adhesion to hydrocarbons (MATH) method as previously described (Vinderola et al., 2004). This method evaluates hydrophobicity by measuring the affinity of microorganisms for organic solvents such as hexane, xylene, or toluene. The *Lactobacillus* isolates were grown overnight in a shaker incubator at 37°C in MRS broth and then harvested by centrifugation at 8000 rpm at room temperature. The cells were washed twice using 1 × PBS and adjusted to an optical density of 0.8–1.0 at A_600 nm_ (A_0_). Next, 1 mL of xylene or hexane was added to each suspension and the mixture was vortexed vigorously for 2 min. After 1 h of incubation at room temperature without shaking and phase separation, the aqueous phase was carefully removed and its absorbance (A_t_) was measured. The hydrophobicity percentage was calculated using the following formula: hydrophobicity (%) = (1 − A_t_/A_0_) × 100.

### Evaluation of auto-aggregation

Auto-aggregation assays were performed as described previously (Zuo et al., 2016). Briefly, *Lactobacillus* isolates were grown overnight in MRS broth at 37°C and harvested by centrifugation at 8000 rpm at room temperature. The cells were then washed twice with 1 × PBS (pH = 7.4), and the resulting bacterial suspensions’ OD was adjusted to 0.8–1.0 at A_600nm_ (A_0_). The bacterial suspension was then incubated for 2 h at 37°C without shaking. The upper phase was removed and the OD was measured (A_t_). Finally, the auto-aggregation percentage was determined using the following formula: Auto-aggregation (%) = (OD A_0_ − OD A_t_ /A_0_) × 100.

### *Lactobacillus* biofilm formation assay

The biofilm formation ability of the *Lactobacillus* isolates was assessed using a previously described protocol with minor modifications (Aoudia et al., 2016). The tests were performed under aerobic and anaerobic conditions. Briefly, *Lactobacillus* isolates were cultured overnight in MRS broth at 37°C in a shaker incubator using glycerol stock to obtain the primary culture. After this, all isolates were sub-cultured to achieve log-phase growth (0.5–0.6 OD at A_600nm_). In each well of a 24-well polystyrene plate, 100 μL of the secondary log phase culture was added to 2 mL of MRS medium and incubated at 37°C for 72 h. After incubation, non-adherent bacteria and the culture medium were removed, and the wells were washed twice with sterile distilled water. Plastic-adhered biofilms were fixed with 1 mL of methanol for 15 min, methanol was removed, and the biofilms were air-dried. Finally, the biofilms were stained by using 200 μL of 0.2% crystal violet in distilled water for 10 min, the excess stain was removed with sterile distilled water, and the stain was extracted from the adherent cells using 500 μL of 0.5 M glacial acetic acid. Absorbance was measured using a microplate reader at A_570nm_.

### Epithelial cell adhesion assay

The adhesion abilities of the *Lactobacillus* isolate to epithelial cells were measured as described previously by Jacobsen et al. with minor modifications (Jacobsen et al., 1999). Madin-Darby bovine kidney (MDBK) epithelial cells were cultured in DMEM medium (DMEM, Gibco) at 37°C in a humidified atmosphere containing 5% CO_2_, and were seeded at a density of 3 × 10^5^ cells/mL per well in 6 well plates (Corning Inc., NY, United States). *Lactobacillus* isolates including *Lactobacillus plantarum* MTCC-2621 control strain was grown in MRS broth (MRS-Broth, Himedia) to an OD_A600nm_ of 0.8, and were labeled with Carboxyfluorescein succinimidyl ester (CFSE) fluorescent dye as per the manufacturer’s instructions (Thermos scientific). Briefly, 3 × 10^7^ bacterial cells were washed twice with 1x PBS (phosphate-buffered saline, pH 7.4) via centrifugation and resuspended in 250 μL of 1x CFSE dye and incubated in a shaker at 37°C, 200 rpm for 40 min. Post-labelling with CFSE dye, bacterial cells were washed again centrifuged with 1X PBS at 4500 g for 10 min, and resuspended in DMEM (without FBS, Antibiotics and Antimycotic solution). CFSE dye labeled *Lactobacillus* were used to infect the MDBK cells at an MOI of 1:10. Infected MDBK cells were incubated for 2 h at 37°C in 5% CO2, and subsequently washed three times with Dulbecco’s PBS buffer (DPBS, Gibco) to remove un-adhered bacteria, and adherent bacterial cells were evaluated by fluorescent microscopy (ZEISS, AXIO Observer 7) following DAPI (#62248, ThermoFisher) counter staining. In parallel, bacterial CFU were measured via plating on to MRS-agar plates. For CFU plating cells were detached via addition of 1 mL solution of 0.25% trypsin–EDTA (Sigma, United States) and incubating for 5-min at room temperature (RT). The detached cells were gently aspirated and mixed repeatedly to make homogenous suspension, and was then serially diluted in MRS medium and plated onto MRS agar plates. After incubation for 24 h at 37°C, the colonies were counted. Data were expressed as the percentage of adhesion = (CFU of adhered bacteria per well /bacterial cells initially added) × 100.

### Hemolytic assay

Overnight grown *Lactobacillus* cultures were streaked onto blood agar media containing 5% goat blood and incubated at 37°C for 24 h. *Listeria monocytogenes* and *S. aureus* were used as positive controls for α- and β-hemolysis, respectively. Clear and colored zones surrounding the colonies were examined. Clear zones indicated beta hemolysis, greenish zones indicated alpha hemolysis, and the absence of zones indicated no hemolysis or gamma hemolysis.

### Measurement of antibiotic susceptibility and the minimum inhibitory concentration (MIC)

Antibiotic susceptibility of the *Lactobacillus i*solates was tested using the broth microdilution method. Nine antibiotics were selected, including β-lactams like ampicillin (0.125–64 mg/L), macrolides like erythromycin (0.0039–2 mg/L), lincosamides like clindamycin (0.0156–8 mg/L), glycopeptides such as vancomycin (0.125–64 mg/L), aminoglycosides like gentamicin (0.031–16 mg/L), kanamycin (0.125–64 mg/L), streptomycin (0.125–64 mg/L), tetracycline (0.125–64 mg/L), and chloramphenicol (0.125–64 mg/L), as recommended by FEEDAP and EFSA panels for human and veterinary importance [EFSA Panel on Additives and Products or Substances used in Animal Feed (FEEDAP), 2012]. Briefly, the isolated *Lactobacillus* were propagated overnight in MRS broth at 37°C to reach an OD (A_600nm_) of 1.0. Microtiter test plates containing 2-fold serially diluted antibiotics in *Lactobacillus* susceptibility medium (LSM-broth) were inoculated at a final inoculum density of 0.001 (A_600nm_), equivalent to 10^5^ CFU/mL. After aerobic incubation at 37°C for 24 and 48 h, MIC values of each antibiotic were visually evaluated two times at 24 and 48 h as the lowest antibiotic concentrations at which no growth was observed. The susceptibility status of strains was interpreted according to the microbiological cut-off values defined by the EFSA Panel on bacterial Feed Additives and Products or Substances used in Animal Feed in relation to antimicrobial resistance (EFSA Panel on Animal Health and Welfare (AHAW) et al., 2021).

### Assessment of antimicrobial activity

The antimicrobial activity of all *Lactobacillus* isolates was analyzed against *ESKAPE* pathogens using an agar well diffusion assay. *ESKAPE* bacteria were grown overnight to an OD (A_600nm_) of 1.0, and 10-fold serial dilutions were made in normal saline. 100 μL of the bacterial inoculum was spread onto nutrient agar plates. Wells (10 mm diameter) were made on agar plates using 1 mL sterile tip bottoms. To extract CFS from *Lactobacillus* isolates, they were grown overnight and centrifuged at 9,000 rpm at 4°C, and supernatants were collected and filter sterilized with a 0.2-micron filter. 100 μL of CFS was added to the wells on each agar plate. To obtain the bacterial cell extract, overnight cultures of *Lactobacillus* were centrifuged at 9,000 rpm and 4°C, and the supernatant was discarded. Cell pellets were washed with 1x PBS (phosphate buffer saline, pH 7.4), and cells were disrupted by homogenization using a bead-beater using 0.1 mm beads in phosphate buffer; finally, following centrifugation cell lysate was filtered through a 0.2-micron filter and the sterile filtrate was used for the antimicrobial assay. After 24 h of incubation at 37°C under aerobic conditions, zone inhibition (mm) was recorded using the zone of inhibition scale (PW297, Himedia).

### *ESKAPE* pathogen biofilm inhibition assay

The anti-biofilm properties of CFS of *Lactobacillus* isolates against *ESKAPE* pathogens were assessed in 24-well plates. Briefly, 500 μL of *ESKAPE* bacterial culture of 0.3 OD (A_600nm_) in LB broth was added to each well along with 500 μL of CFS (in MRS media). 1: 1 mixture of L.B broth and MRS was used as a negative control. *ESKAPE* bacterial culture (0.3 OD) without CFS was used as a positive control. The microtiter plates were incubated at 37°C for 48 h to allow for biofilm formation. Subsequently, biofilm formation was measured as described in the ‘Biofilm formation assay’ section.

### Statistical analysis

GraphPad Prism 9 was used for the preparation of the graphs and to perform the statistical analysis. For comparison of group means, One-Way ANOVA or t-test was performed where ever applicable, and differences were considered significant when *p* < 0.05. All the results are shown as the mean ± SD unless otherwise described in the corresponding figure legends.

## Results

### Isolation, identification, and biochemical characterization of *Lactobacillus* from caprine gut

Gastrointestinal (GI) infections in livestock ruminants not only have significant implications for animal health and productivity but also impact public health as a source of zoonotic enteric and other diseases in humans. Resident probiotic bacteria in the small intestine of domestic goats may have evolved niche-specific colonization and host-favoring properties that promote the maintenance of enteric homeostasis and antagonize the growth of pathogens in the gut. To exploit the beneficial properties of such enteric probiotic *Lactobacillus*, we first collected small intestinal (jejunum) tissues from goats and subjected them to a standard MRS media-based *Lactobacillus* isolation method (De Man et al., 1960; Dave and Shah, 1996). After several rounds of isolation of potential—*Lactobacillus* colonies with creamy white color on MRS agar, we selected 54 colonies for further confirmation by *Lactobacillus* genus-specific PCR using 16S-rRNA gene-specific primers, Forward-R16-1 and Reverse-LbLMA1, as described previously by Dubernet et al. (2002) (Supplementary Table S1). Out of 54 colonies, we selected 20 that showed an expected amplicon of ~250 bp (Supplementary Figure S1A). Furthermore, these 20 isolates were subjected to a second round of PCR and amplicon sequencing targeting 16S-rRNA V1-V3 region and the 16S-23S Intergenic Spacer Region (ISR) for species identification (Weisburg et al., 1991; Gurtler and Stanisich, 1996; Berthier et al., 1998; Turner et al., 1999). Two separate primer pair sets were used to amplify ~509 bp (Supplementary Figure S1B) and ~ 565 bp regions (Supplementary Figure S1C) from the 16S-rRNA-V1-V3 region and 16S-23S ISR using 16S(8–27)-F and V3(519–536)-R and 16S(ISR)-F and 23S(ISR)-R primer pairs, respectively (Supplementary Table S1). Out of the 20 *Lactobacillus* -like colonies that were subjected to PCR, followed by Sanger sequencing and NCBI BLAST-based nucleotide homology analysis, 12 isolates showed the closest identity to *Lactobacillus* spp., while the other colonies were found to belong to either *Enterococcus* spp. or *Acetobacter* spp. (Table 1). All sequences were submitted to NCBI Bio-project No. PRJNA985412, PRJNA986841, PRJNA986842. Among the 13 *Lactobacillus* species, 5 were identified as *Lp. plantarum*, 2 as *L. salivarius*, 2 as *L. crispatus*, 2 as *L. amylovorous*, 1 as *L. kitasatonis*, and the other as *L. jhonsonii* (Table 1).

**Table 1:** 16 s-rRNA gene V1-V3 region and 16 s-23 s ISR amplicon sequencing and identification of *Lactobacillus* isolates.

S. No.	Isolate No.	Primer set-1: 16 s (ISR)-F & 23S (ISR)-R			Primer set-2: 16 s (8–27)-F & V3 (519–536)-R		
		Similarity to organism	% Identity	GenBank Accession No	Similarity to organism	% Identity	GenBank Accession No
1	GJ002C02	*Lactobacillus johnsonii strain G2A*	100.00%	CP040854.1	*Lactobacillus johnsonii strain 1,696*	100.00%	MT597568.1
2	GJ007C03	*Lactobacillus salivarius strain IBB3154*	99.84%	CP027644.1	*Lactobacillus salivarius strain ABRIIN27*	100.00%	MG547723.1
3	GJ008C03	*Enterococcus faecium strain FS86*	99.82%	CP053704.1	*Enterococcus faecium strain So010*	100.00%	OQ940310.1
4	GJ010C02	*Lactobacillus plantarum strain SN13T*	100.00%	AP019815.1	*Lactobacillus plantarum sub*sp. *plantarum strain 28.9 E*	99.53%	MH924330.1
5	GJ003C13	*Lactobacillus crispatus strain DC21.1*	99.84%	CP039266.1	*Lactobacillus crispatus strain 3,019*	99.77%	MT613437.1
6	GJ003C20	*Enterococcus faecalis strain 11,154,707*	97.63%	CP046111.1	*Enterococcus faecalis strain unknown 15*	100.00%	MG751354.1
7	GJ005C01	*Lactobacillus plantarum SN13T DNA*	99.84%	AP019815.1	*Lactobacillus plantarum strain TM2*	99.77%	OQ727427.1
8	GJ009C10	*Lactobacillus plantarum SN13T DNA*	100.00%	AP019815.1	*Lactobacillus plantarum strain WWP-7*	99%	MN372129.1
9	GJ011C03	*Lactiplantibacillus plantarum strain IRG1*	99.84%	CP025690.1	*Lactobacillus sp. strain G5*	99.77%	MK971762.1
10	GJ001C06	*Lactobacillus amylovorus strain 30SC*	98.18%	CP002559.1	*Lactobacillus amylovorous 05-1A07 16S rRNA gene*	89.34%	KX688702.1
11	GJ002C13	*Enterococcus faecalis strain 11,154,707*	97.63%	CP046111.1	*Enterococcus faecalis URL, 16 s rRNA partial seq*	99.79%	KY962967.1
12	GJ007C14	*Lactobacillus salivarius JCM 1046*	100.00%	CP007646.1	*Lactobacillus salivarius 3,316, 16 s rRNA partial seq*	99.56%	MT613610.1
13	GJ008C11	*Acetobacter pasteurians CICC 22518*	94.68%	CP039846.1	*Acetobacter pasteurians AS1.41, 16 s Rrna, partial seq*	98.77%	KY283052.1
14	GJ010C06	*Lactobacillus plantarum SNI3T DNA*	99.53%	AP019815.1	*Lactobacillus plantarum R22, 16 s rRNA, partial seq*	98.57%	MG841153.1
15	GJ003C03	*Enterococuus faecalis JY32*	99.43%	CP045045.1	*Enterococcus faecalis Unknown, 16 s rRNA partial seq*	99%	MG751354.1
16	GJ003C06	*Enterococcus faecalis JY32*	99.58%	CP045045.1	*Enterococcus faecalis TEM75*	100.00%	HT539138.1
17	GJ003C09	*Lactobacillus crispatus DC21.1*	100.00%	CP039266.1	*Lactobacillus crispatus 7,618, 16 s rRNA partial seq*	98.95%	MTS16168.1
18	GJ005C06	No significant similarity	–	–	*Enterococcus faecalis strain A-TSB-6*	99.76%	JX290561.1
19	GJ011C06	*Lactobacillus amylovorus strain 30SC*	85.02%	CP002559.1	No significant similarity	–	–
20	GJ001C03	No significant similarity	–	–	*Lactobacillus kitasatonis strain JCM 1039*	99.60%	MN587975.1

For species identification PCR was performed targeting the 16S-rRNA V1-V3 region and 16S-23S Intergenic Spacer Region (ISR), and two separate primer pair amplified ~ 509 bp, and ~ 565 bp. The amplified PCR products were purified and sequenced via Sanger sequencing using the forward primers of each PCR described above. The trimmed sequences were subjected to the NCBI-BLAST analysis with default parameters for identifying the closest bacterial species with maximum percentage identity.

**Figure 1:**
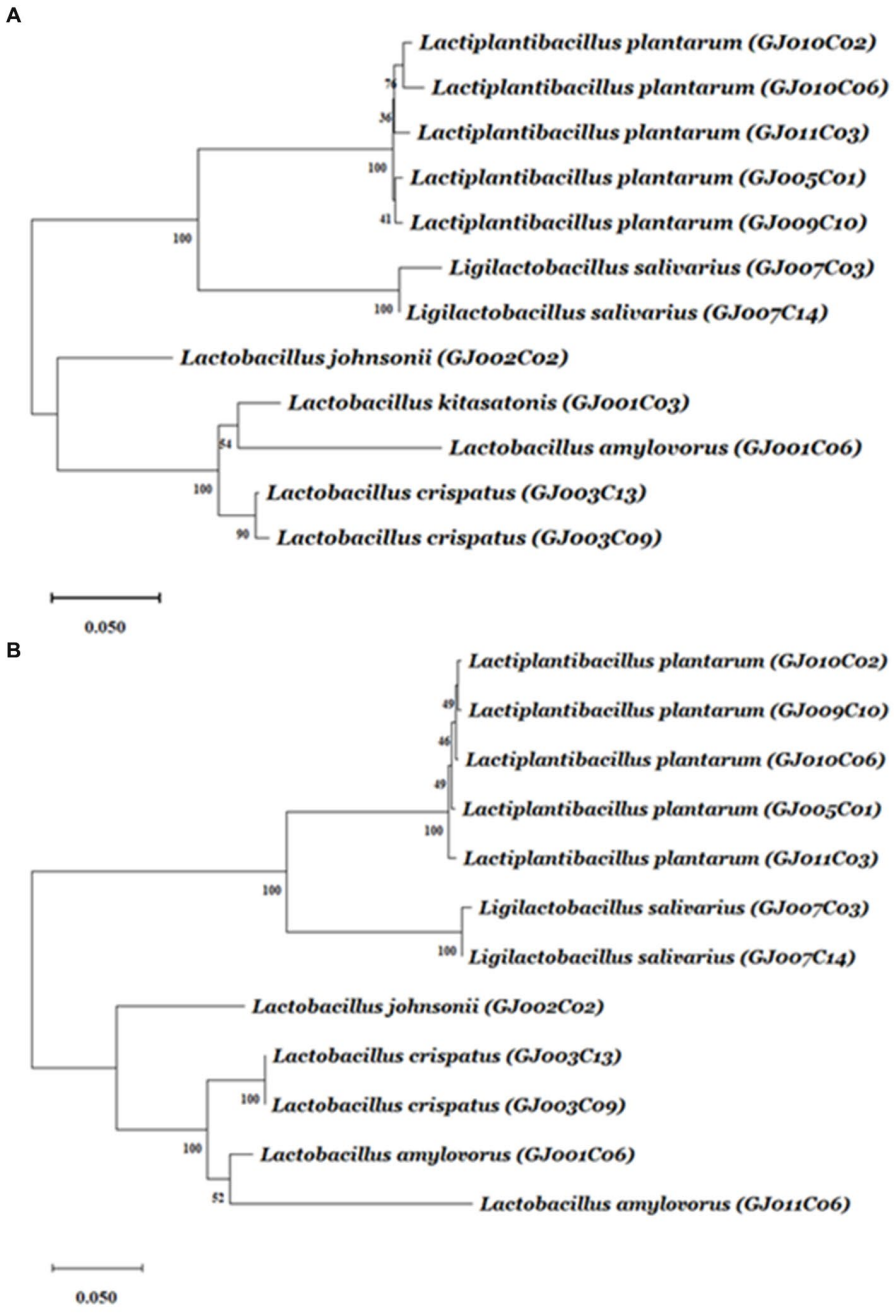
The dendrogram tree of *Lactobacillus* isolates based on 16S rRNA gene V1-V3 region and 16S-23S ISR sequences. The amplicons from the 16S rRNA gene V1-V3 region and 16S-23S ISR were sequenced using the primers 16S(8-27)-F and 16S(ISR)-F, respectively. From each isolate, either of the sequences was aligned using ClustalW with default parameters. The alignment results were then used to find out best fitted nucleotide-substitutional model. The Kimura 2-parameter model and Tamura 3-parameter model are used for the V1-V3 region, and ISR region, respectively. The neighbor-joining method (bootstrap test −1,000 replicates) was used to generate the hierarchical clustering-based dendrogram trees based on **(A)** 16S rRNA gene V1-V3 region, and **(B)** 16S-23S ISR region. A unit of the distance scale represents the percentage of differences between two sequences. Evolutionary analyses were conducted using MEGA11 software.

Subsequently, phylogenetic analysis of the 12 *Lactobacillus* isolates using either of the sequences of the 16S rRNA-V1-V3 region (Figure 1A) or 16S-23S ISR (Figure 1B) via hierarchical clustering using the neighbor-joining method exhibited close evolutionary relationships and relatedness among the caprine gut-derived *Lactobacillus* isolates. As shown in the dendrograms in Figure 1, isolates belonging to the same species of *Lactobacillus* were mapped to be closely related by both methods. Moreover, isolates from the same animal exhibited the closest phylogenetic relationship.

Furthermore, 12 selected *Lactobacillus* isolates were subjected to a series of biochemical tests to meet the criteria for *Lactobacillus* species, as described in *Bergey’s Manual of Determinative Bacteriology* (Bergey, 1994). These include Gram staining, catalase test, nitrate reduction (NR) test, indole test, and Vogues–Proskauer (VP) tests. All 12 isolates showed gram-positive staining, of which six were identified as catalase-negative, while the remaining six were catalase-positive. All 12 *Lactobacillus* isolates showed negative results for the indole test, nitrate test, and VP test (except one showing weak + VP test) (Table 2). These features of the *Lactobacillus* isolates corroborate the standard characteristics of *Lactobacillus* spp. Further, 6 catalase negative *Lactobacillus* isolates were selected for further characterization by carbohydrate fermentation test.

**Table 2:** Biochemical characterization of *Lactobacillus* isolates.

S. No.	*Lactobacillus* isolates	Gram’s stain	Catalase test	Nitrate test	Indole test	VP test
1.	*Lp. plantarum (MTCC-2621)*	+	−	−	−	−
2.	*L. kitasatonis (GJ001C03)*	+	+	−	−	−
3.	*L. amylovorous (GJ001C06)*	+	+	−	−	weak +
4.	*L. jhonsonii (GJ002C02)*	+	+	−	−	−
5.	*L. crispatus (GJ003C09)*	+	−	−	−	−
6.	*L. crispatus (GJ003C13)*	+	+	−	−	−
7.	*Lp. plantarum (GJ005C01)*	+	−	−	−	−
8.	*Lg. salivarius (GJ007C03)*	+	−	−	−	−
9.	*Lg. salivarius (GJ007C14)*	+	+	−	−	−
10.	*Lp. plantarum (GJ009C10)*	+	−	−	−	−
11.	*Lp. plantarum (GJ010C06)*	+	−	−	−	−
12.	*Lp. plantarum (GJ011C03)*	+	−	−	−	−
13.	*L. amylovorous (GJ011C06)*	+	+	−	−	−

+, positive for the given test; −, negative for the given test; weak +, slight positive reaction. *Lp. plantarum (MTCC-2621)* used as a positive control.

Each *Lactobacillus* isolate was screened for Gram staining, catalase activity, indole test, for the production of indole from tryptophan; nitrate test for the reduction of nitrate to nitrite; and Vogues Proskauer (VP) test to determine the presence of acetyl methyl carbinol after glucose fermentation. Six of the Gram-positive and catalase-negative *Lactobacillus* isolates were selected for further analysis.

Probiotic *Lactobacillus* with complex sugar fermentation capability is important for their use in ruminant feed because ruminants rely on microbial fermentation of complex sugars in their digestive process (Matthews et al., 2019). Here, we assessed the carbohydrate fermentation ability of these six *Lactobacillus* isolates on 10 different carbohydrate sugars, namely, monosaccharides (Xylose, Arabinose, Galactose, Mannose), disaccharides (Cellobiose, Maltose, Melibiose, Sucrose, Trehalose), and trisaccharide (Raffinose) using a Hilacto™ Identification kit for *Lactobacillus* which also includes catalase and esculin tests. All six *Lactobacillus* isolates utilized sugars at different rates of hydrolysis, indicating varied carbohydrate fermentation capabilities (Table 3; Supplementary Figure S2). Furthermore, the *Lactobacillus* isolates displayed a positive esculin test, which is a typical feature of *Lactobacillus* spp. confirming their ability to break down complex coumarin glycoside-esculin into glucose and esculetin in the presence of β-glucosidase or esculinase enzyme (Chuard and Reller, 1998).

**Table 3:** *Lactobacillus* isolates exhibit differential carbohydrate fermentation ability.

*Lactobacillus* isolates	Esculin	Catalase	Xylose	Cellobiose	Arabinose	Maltose	Galactose	Mannose	Melibiose	Raffinose	Sucrose	Trehalose
*Lp. plantarum (MTCC-2621)*	+	−	+	+	+	+	+	+	+	+	+	+
*L. crispatus (GJ003C09)*	+	−	+	+	+	+	+	+	+	weak +	+	+
*Lp. plantarum (GJ005C01)*	+	−	+	+	+	+	+	+	+	+	+	+
*Lg. salivarius (GJ007C03)*	+	−	+	+	+	+	+	+	weak +	weak +	+	+
*Lp. plantarum (GJ009C10)*	+	−	+	+	+	+	+	+	weak +	weak +	+	+
*Lp. plantarum (GJ010C06)*	+	−	+	+	+	+	+	+	+	+	+	+
*Lp. plantarum (GJ011C03)*	+	−	+	+	+	+	+	+	weak +	+	+	+


+, positive for the given test; −, Negative for the given test; weak +, slight positive reaction. *Lp. plantarum (MTCC-2621)* used as a positive control.

The table depicts the carbohydrate fermentation ability of selected 6 *Lactobacillus* isolates. The carbohydrate fermentation was measured via colorimetric identification based on the principle of pH change and substrate utilization for the genus *Lactobacillus*. All isolates were found esculin positive, catalase-negative, and capable of fermenting different carbohydrate sugars with varied abilities.

### Caprine gut-derived *Lactobacillus* isolates exhibited acid stress and bile salt tolerance

**Figure 2:**
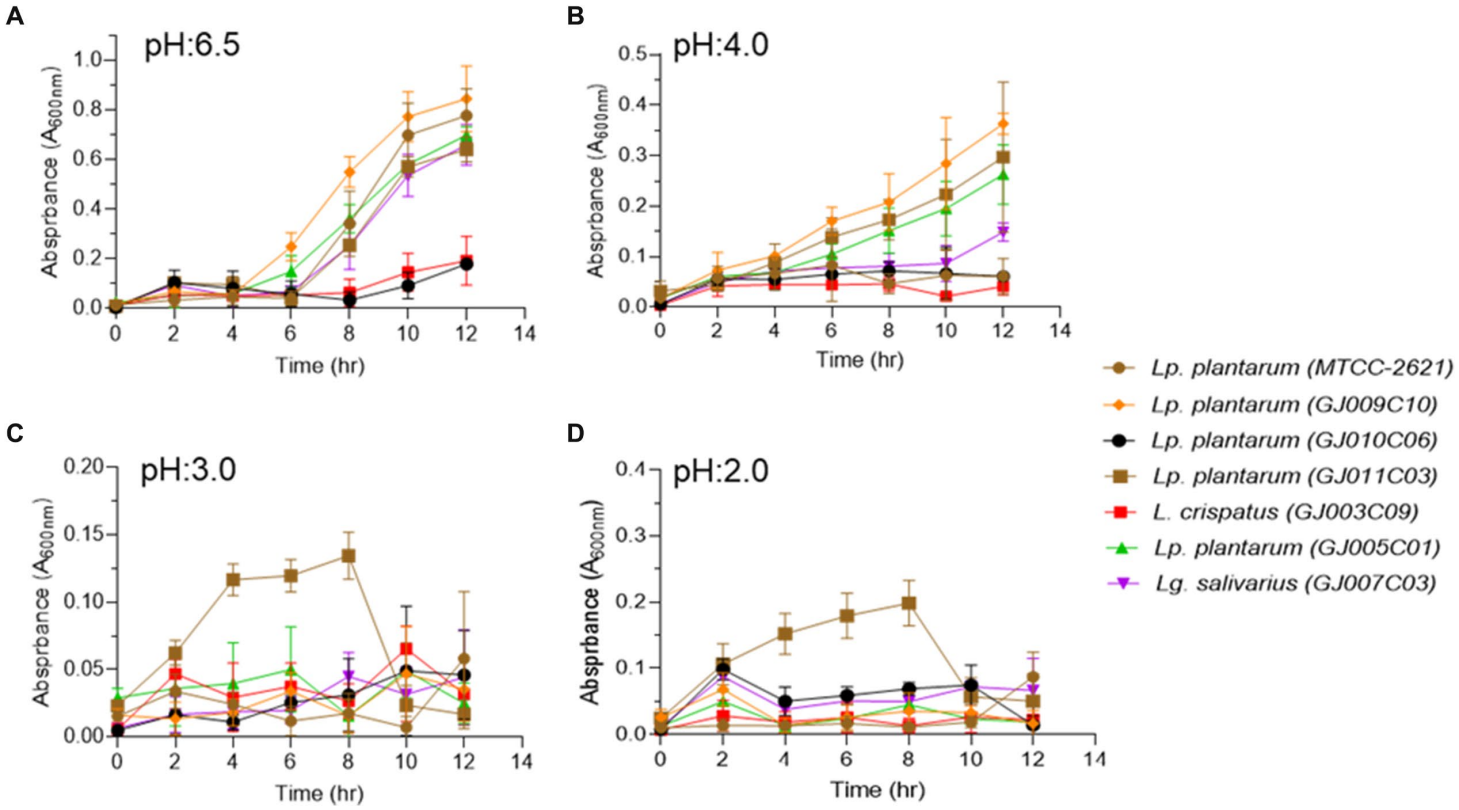
Profiling of the ability of *Lactobacillus* isolates to pH stress. Tolerance of *Lactobacillus* isolates to lower pH were assessed in three different conditions in addition to the MRS media (pH 6.5). Absorbance (A_600nm_) was taken at an interval of 2 h and till 12 h post-inoculation. Each connecting point in the line graphs depicts the mean ± SD of the absorbance values at each time point indicating the growth of *Lactobacillus* isolates at **(A)** pH 6.5, **(B)** pH 4.0, **(C)** pH 3.0, and **(D)** pH 2.0 conditions. *Lp. plantarum (MTCC-2621)* was used as positive control.

The ability to tolerate harsh conditions, such as low pH or acidic environment in the stomach, and sustenance in the high bile salt concentration in the initial part of the small intestine are the major determinants for the survival of probiotic bacteria in the gastrointestinal tract (Chou and Weimer, 1999). Each of the six selected *Lactobacillus* isolates displayed different degrees of sensitivity towards pH and bile salt stress, as evidenced by their differential growth patterns monitored over a period of 12 h at different pH (2.0, 3.0, 4.0, and 6.5) and bile salt (0.5, 1.0, and 2.0%)-containing media. In a non-acidic environment at pH 6.5, four of the *Lactobacillus* isolates exhibited typical sigmoidal bacterial growth, except the GJ010C06 and GJ003C09 isolates that displayed longer lag phases with minimal growth (Figure 2A). At pH 4.0, half of the *Lactobacillus* isolates exhibited moderate growth, while the other half displayed reduced or stalled growth (Figure 2B). Nevertheless, all the isolates, including GJ010C06 and GJ003C09, survived acid stress at lower pH values of 2.0, 3.0, while the GJ011C03 strain exhibited the highest growth in these low pH environments (Figures 2C,D).

**Figure 3:**
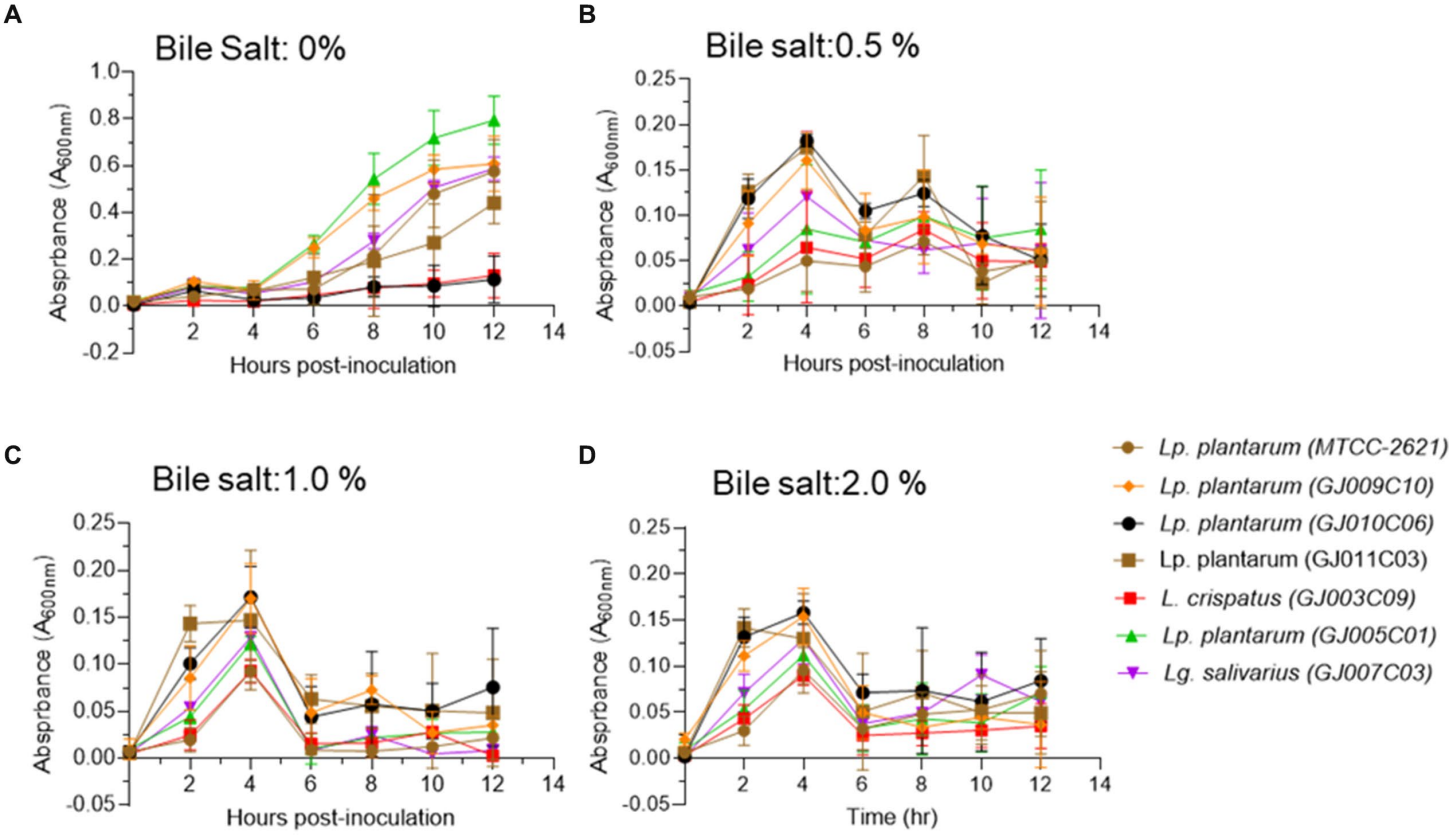
Bile tolerance profile of *Lactobacillus* isolates. Bile salt tolerance of *Lactobacillus* isolates was assessed in three different concentrations in addition to the standard MRS media. Absorbance (A_600nm_) was taken at an interval of 2 h and till 12 h post-inoculation. Each connecting point in the line graphs depicts the mean ± SD of the absorbance values at each time point indicating the growth of *Lactobacillus* isolates in **(A)** 0% bile salt, **(B)** 0.5%, **(C)** 1%, and **(D)** 2% w/v conditions. *Lp. plantarum (MTCC-2621)* was used as positive control.

When bile salts were added to the growth medium at three different concentrations (0.5, 1, and 2% w/v), all selected *Lactobacillus* isolates exhibited suppressed growth compared with the standard growth media (Figure 3A). However, they were able to endure the salt stress for up to 12 h in medium containing 0.5% bile salt (Figure 3B). In media containing 1.0 and 2.0% bile salt, after an initial normal growth until 4 h post-inoculation, the *Lactobacillus* isolates displayed a sharp reduction in culture absorbance, followed by a plateau phase of growth (Figures 3C,D). Among the six *Lactobacillus* isolates, GJ009C10, GJ010C06, and GJ011C03 maintained higher culture absorbance levels than the other three *Lactobacillus* isolates.

### Cell surface hydrophobicity and autoaggregation properties of *Lactobacillus* isolates

**Figure 4:**
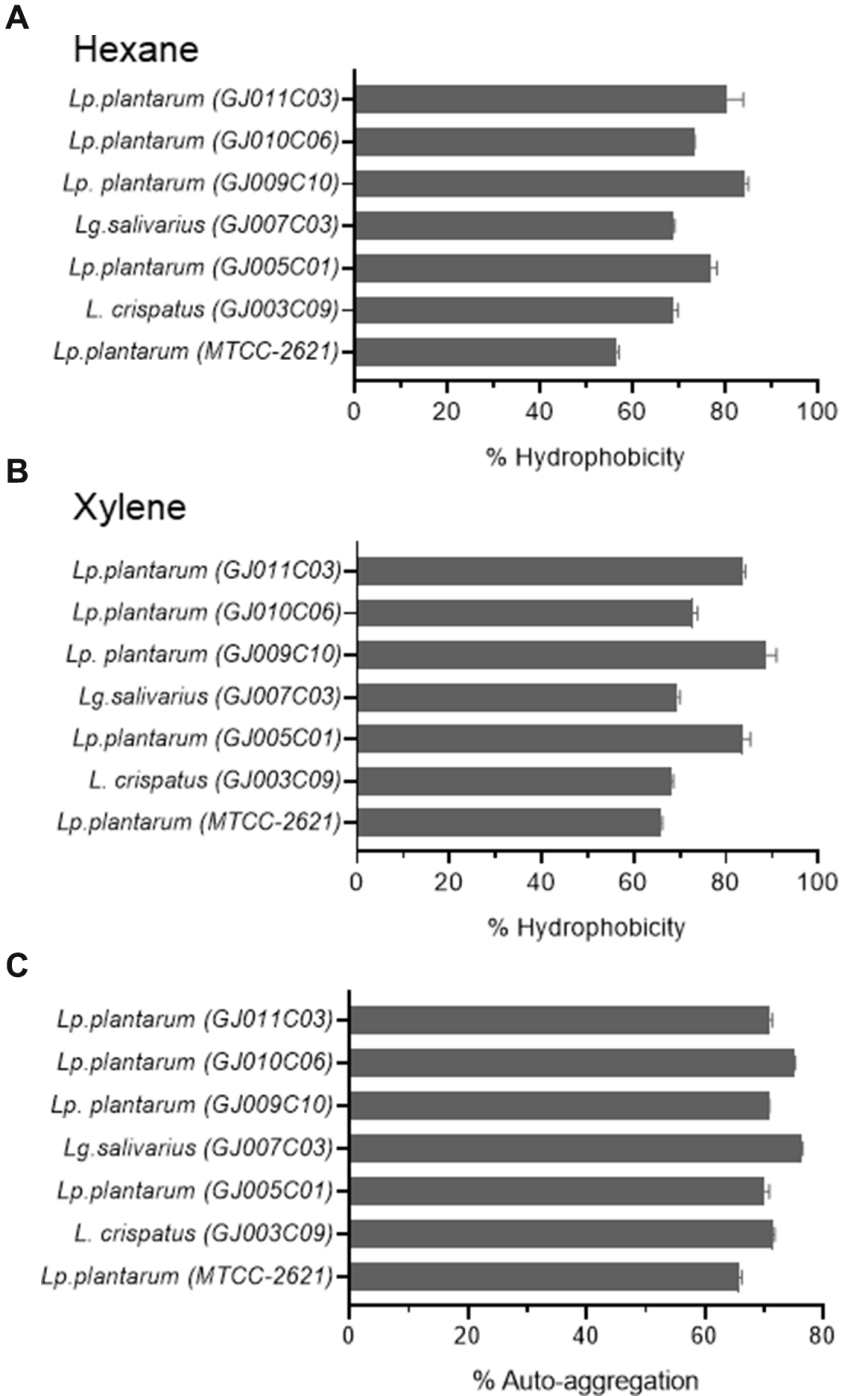
Cell surface properties of selected *Lactobacillus* isolates. The figure depicts the **(A,B)** % Hydrophobicity and **(C)** % Autoaggregation of *Lactobacillus* isolates. The % hydrophobicity was measured using the microbial adhesion to hydrocarbons (MATH) using **(A)** hexane, and **(B)** xylene as organic solvents as described in the Materials & methods section. **(C)** Autoaggregation is measured by evaluating the physical clumping ability of the *Lactobacillus* isolates in PBS solution as described earlier. The data is the Mean ± SD of triplicate experiments. *Lp. plantarum (MTCC-2621)* was used as positive control.

Probiotic bacteria need to adhere to the mucus in the gut to survive, and their ability to do so is crucial for competition with harmful bacteria (Tuomola et al., 2001; Ohland and MacNaughton, 2010). Adhesion to the intestinal wall involves both nonspecific and specific interactions facilitated by different cell components (Magnusson et al., 1985; Krausova et al., 2019). Cell surface hydrophobicity, measured by the microbial adhesion to hydrocarbon method (MATH), is an important factor for adhesion capacity and is considered a pre-test for epithelial cell adhesion ability (Vinderola et al., 2004). Similarly, auto-aggregation is a process in which bacteria physically interact with each other with the help of cell surface components, such as proteins, carbohydrates, and lipoteichoic acid. Auto-aggregation of probiotics is necessary for adherence to the gut lining and acts as a barrier against undesirable bacteria (Tuo et al., 2013). These characteristics provide advantages for probiotics to colonize the gut. Hydrophobicity was assessed using two organic compounds: Hexane (Figure 4A) and Xylene (Figure 4B). All of the *Lactobacillus* isolates exhibited significant adhesion rates (% hydrophobicity) of over 60%. *Lactobacillus* isolates GJ009C10 and GJ005C01 exhibited >80% adhesion rates. Although both organic solvent-based techniques showed a comparable trend in % Hydrophobicity, the xylene-based method yielded higher values than the hexane-based method. The selected *Lactobacillus* isolates also displayed a recommended range of 60 to 80% auto-aggregation percentage, with GJ007C03 and GJ010C06 as top performers (Figure 4C). Interestingly, all the *Lactobacillus* isolates exhibited comparatively higher % Hydrophobicity and % Autoaggregation compared to the *Lp. plantarum (MTCC-2621)* control strain.

### *Lactobacillus* isolates formed more biofilms in the anaerobic environment

**Figure 5:**
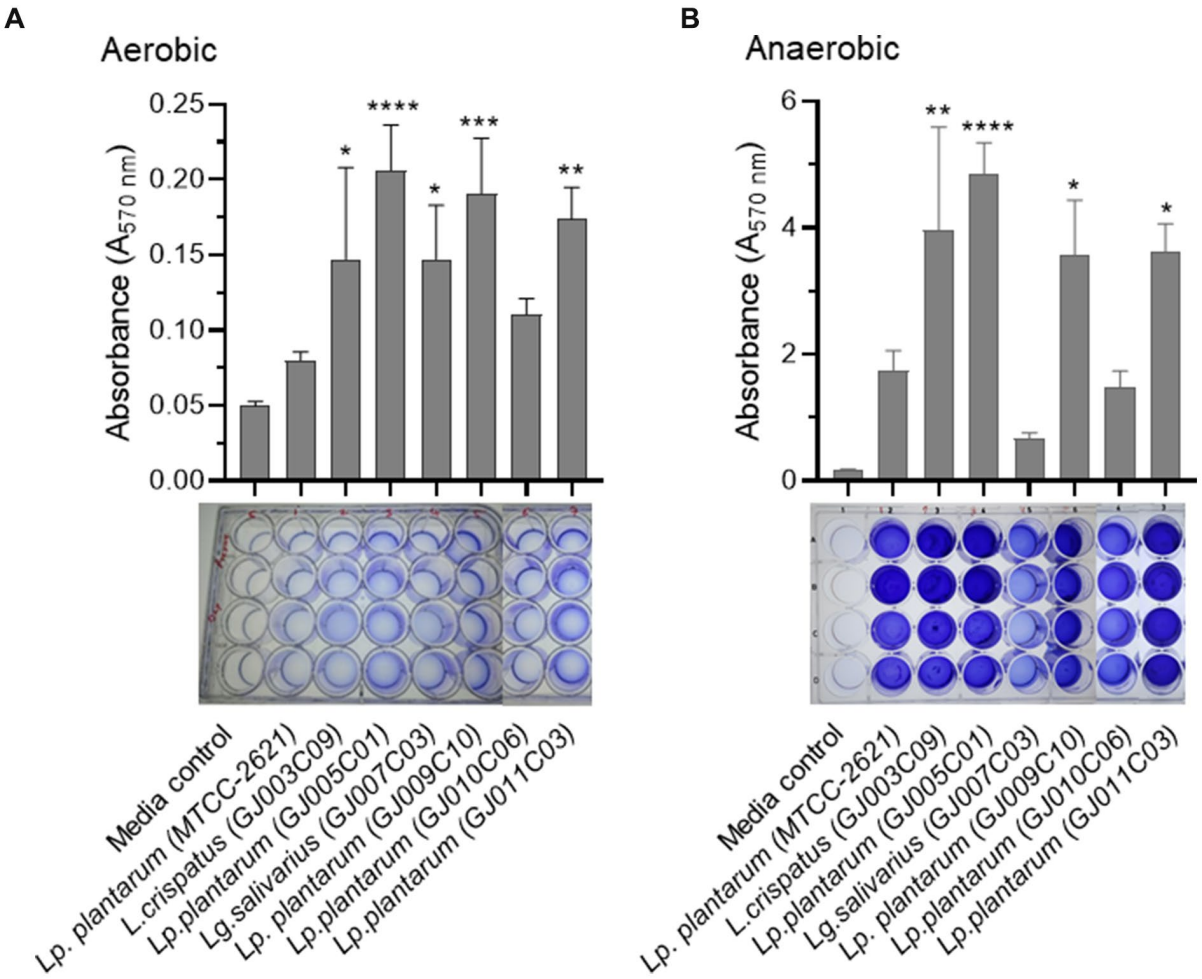
Biofilm formation by *Lactobacillus* isolates in aerobic and anaerobic environments. The biofilm formation ability of *Lactobacillus* isolates was assessed using both **(A)** aerobic, and **(B)** anaerobic growth conditions. The assay was performed in 24-well polystyrene plates. *Lactobacillus* isolates were cultured in static conditions in MRS media at 37°C for 72 h. Subsequently, quantitative measurement of biofilm was performed using the standard crystal violet-based assay. The bar diagrams depict the Mean ± SD of the absorbance (A_570nm_) values from triplicate wells. The experiment was performed twice. One-way ANOVA was performed to compare the mean differences of each *Lactobacillus* isolate compared to the *Lp. plantarum (MTCC-2621)* control strain. *p < 0.05, **p < 0.01, ***p < 0.001, ****p < 0.0001. All *Lactobacillus* isolates produced more biofilm in the anaerobic conditions compared to the aerobic environment.

A promising approach for the control of pathogenic bacterial biofilms is the use of probiotics to colonize epithelial surfaces and counteract the proliferation of other bacterial species via competitive exclusion (Salas-Jara et al., 2016). Probiotic biofilms can promote their self-colonization and longer persistence in the intestinal mucosa, and *Lactobacillus* species produce more robust biofilms than other species (Kubota et al., 2008). Here, we tested the biofilm formation ability of *Lactobacillus* isolates under both aerobic (Figure 5A) and anaerobic conditions (Figure 5B) as they are facultative-anaerobic in nature, and both conditions prevail in the gastrointestinal tract. While all selected *Lactobacillus* isolates formed biofilms under both aerobic and anaerobic conditions, a significantly greater mass of biofilms was formed under anaerobic conditions. The most abundant biofilm-forming *Lactobacillus* isolates under both aerobic and anaerobic conditions were GJ005C01, GJ009C10, GJ003C09, and GJ011C03.

### *Lactobacillus* isolates possess epithelial cell adhesion properties

**Figure 6:**
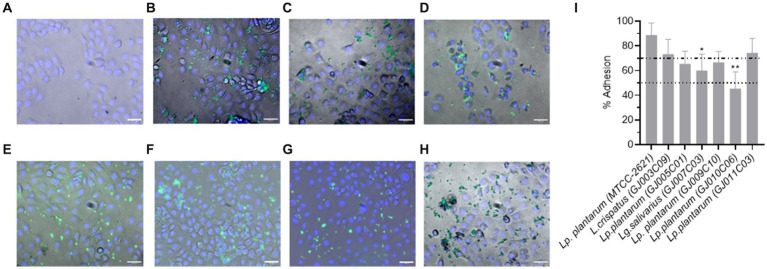
Cell adhesion ability of *Lactobacillus* isolates. Representative fluorescence microscopic images of MDBK cells after 2-h of incubation with fluorescently labeled probiotic isolates at an MOI of 1:10. **(A)**. MDBK cells only (Negative control), **(B–H)** MDBK cells incubated with **(B)** MTCC-2621 (Positive control), **(C)** GJ003C09, **(D)** GJ005C01, **(E)** GJ007C03, **(F)** GJ011C03, **(G)** GJ009C10, and **(H)** GJ010C06. Scale bars: 100 μm for 20X objectives. **(I)** CFU of all the *Lactobacillus* isolates was measured by bacteriological plating from triplicate wells and % adhesion was calculated and presented as a bar diagram depicting mean ± SD. The top and bottom dotted lines represent the boundaries between the strong and moderate, and moderate and low adherent *Lactobacillus* isolates. ANOVA test was performed to compare the mean of *Lactobacillus* isolates to that of *Lp. plantarum (MTCC-2621)* control strain. *p < 0.05; **p < 0.01.

The adhesion ability of *Lactobacillus* isolates onto the MDBK cells was determined by fluorescence microscopy and CFU counting. Fluorescence microscopy showed CFSE labeled green fluorescent bacteria bound to cell surface, however variations in the adhesion was apparent across the *Lactobacillus* isolates (Figures 6A–H). CFU counts of the adhered bacteria (Supplementary Figure S3) and derivation of the % adhesion ability (Figure 6I) supported the microscopic findings. The percentage of adhesion varied from 45.5% (GJ010C06) to 74.4% (GJ011C03) among the *Lactobacillus* isolates. *Lactobacillus* isolates were then categorized based on their % adhesion as described previously (Jacobsen et al., 1999): strongly adhesive (>70%, GJ011C03 and GJ003C09), moderately adhesive (50–70%, GJ009C10, GJ007C03 and GJ005C01), and weakly adhesive (<50%, GJ010C06). The % adhesion ability of GJ003C09, GJ009C10, GJ011C03, and GJ005C01 were statistically similar to the MTCC control strain, while GJ007C03 and GJ010C06 showed significantly low % adhesion (*p* < 0.05 and *p* < 0.01, respectively).

### Caprine gut-derived *Lactobacillus* isolates are non-hemolytic

While *Lactobacillus* belong to the GRAS category of bacteria and are typically safe for consumption, newly isolated LAB strains must be proven to be devoid of any toxic effects, such as hemolytic properties, which can lead to serious health problems including anemia and kidney damage (Naidu et al., 2012; Tanaka et al., 2023). To ensure that the caprine gut-derived novel *Lactobacillus* isolates were safe, we performed hemolytic profiling for α-hemolysis (Supplementary Figure S4A) and β-hemolysis (Supplementary Figure S4B), wherein *L. monocytogenes* and *S. aureus* were used as positive controls for α- and β-hemolysis, respectively (Mogrovejo et al., 2020). None of the selected caprine gut-derived *Lactobacillus* isolates showed a hemolytic effect, highlighting their safety for consumption (Supplementary Figure S4C).

### Caprine gut-derived *Lactobacillus* isolates are susceptible to a panel of major antibiotics

Antimicrobial resistance is a significant safety concern when evaluating the use of *Lactobacillus* as feed additive and therapeutic [European Food Safety Authority (EFSA), 2005]. The food chain has been identified as a major route of drug-resistant bacterial transmission between animals and humans, highlighting the need to monitor the safety of *Lactobacillus* used in animal nutrition (Brashears et al., 2005). Here, we screened the caprine gut-derived *Lactobacillus* isolates against nine antibiotics, including ampicillin (0.125–64 mg/L), erythromycin (0.0039–2 mg/L), clindamycin (0.0156–8 mg/L), vancomycin (0.125–64 mg/L), gentamicin (0.031–16 mg/L), kanamycin (0.125–64 mg/L), streptomycin (0.125–64 mg/L), tetracycline (0.125–64 mg/L), and chloramphenicol (0.125–64 mg/L) using the broth dilution-based minimum inhibitory concentration (MIC) method in LSM media using the microbiological breakpoint (BP), or cut-off values recommended by the FEEDAP document, and EFSA [EFSA Panel on Additives and Products or Substances used in Animal Feed (FEEDAP), 2012]. The MIC for all LAB isolates were measured using a U-bottom microplate-based growth assay followed by visual observation at 24 h post-inoculation (Table 4). All isolated *Lactobacillus* strains were found to be susceptible to nine of the antibiotics tested, except vancomycin, and the MIC was below the epidemiological cutoff suggested by EFSA [EFSA Panel on Additives and Products or Substances used in Animal Feed (FEEDAP), 2012].

**Table 4:** Susceptibility of *Lactobacillus* isolates to EFSA-specified list of antibiotics.

	Ampicillin	Vancomycin	Gentamycin	Kanamycin	Streptomycin	Erythromycin	Clindamycin	Tetracycline	Chloramphenicol
*Lactobacillus* sp. EFSA specified MIC cut off (mg/L)	1–4	2, n.r.	8–32	16–64	16–64, n.r.	1	1–2	4–32	4–8
*Lp. Plantarum (MTCC-2621)*	2.00	>128	0.125	4–8	4	0.031	0.015	16	2
*L. crispatus (GJ003C09)*	0.25	>128	0.062	1–2	1–2	0.312	0.031	2–4	2–4
*Lp. plantarum (GJ005C01)*	0.50	>128	0.250	8	1	0.062	0.062	8–16	2
*Lg. salivarius (GJ007C03)*	0.50	>128	0.250	4–8	2	0.062	0.062	8	1
*Lp. plantarum (GJ009C10)*	0.25	>128	0.125	4	1	0.062	0.062	4–8	2
*Lp. plantarum (GJ010C06)*	1.00	>128	0.125	4	1	0.312	0.031	4–8	0.5
*Lp. plantarum (GJ011C03)*	0.25	>128	0.125	4	1	0.312	0.062	4	4

n.r., not required; MIC, Minimum inhibitory concentration; EFSA, European Food Safety Authority; *Lp. plantarum (MTCC-2621)* used as a positive control.

The antibiotic susceptibility of the *Lactobacillus* isolates was tested for 9 antibiotics using broth micro-dilution method as recommended by FEEDAP and EFSA panel for human and veterinary importance. MIC values of each antibiotic were visually evaluated at 24 and 48 h as the lowest antibiotic concentrations at which no growth was observed. Experiments were performed thrice. *Lp. plantarum (MTCC-2621)* was used as a reference *Lactobacillus* strain.

### Probiotic *Lactobacillus* isolates limit growth and biofilm formation by *ESKAPE* pathogens

**Figure 7:**
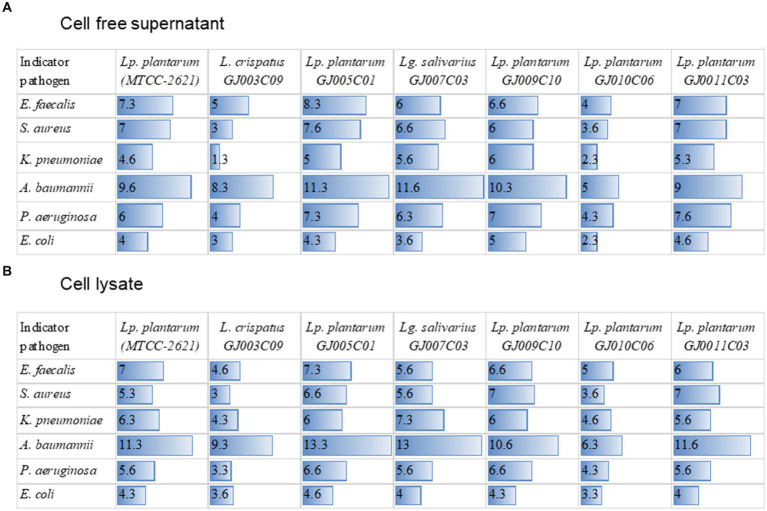
Zone of inhibition of *ESKAPE* pathogen growth by cell-free supernatant and cell-lysates of LAB isolates. The figure depicts the zone of inhibition radius from agar well diffusion assay. The zone of inhibition was recorded with the zone of inhibition scale (PW297, Himedia) following 24 h of incubation after the addition of sterile-filtered **(A)** cell-free culture supernatants, and **(B)** cell lysate of *Lactobacillus* isolates to the agar-wells. The horizontal bar depicts the Mean (values embedded) of the zone of inhibition radius of three experiments (mm) excluding the diameter of the agar well; *Lp. plantarum (MTCC-2621)* used as a positive control.

**Figure 8:**
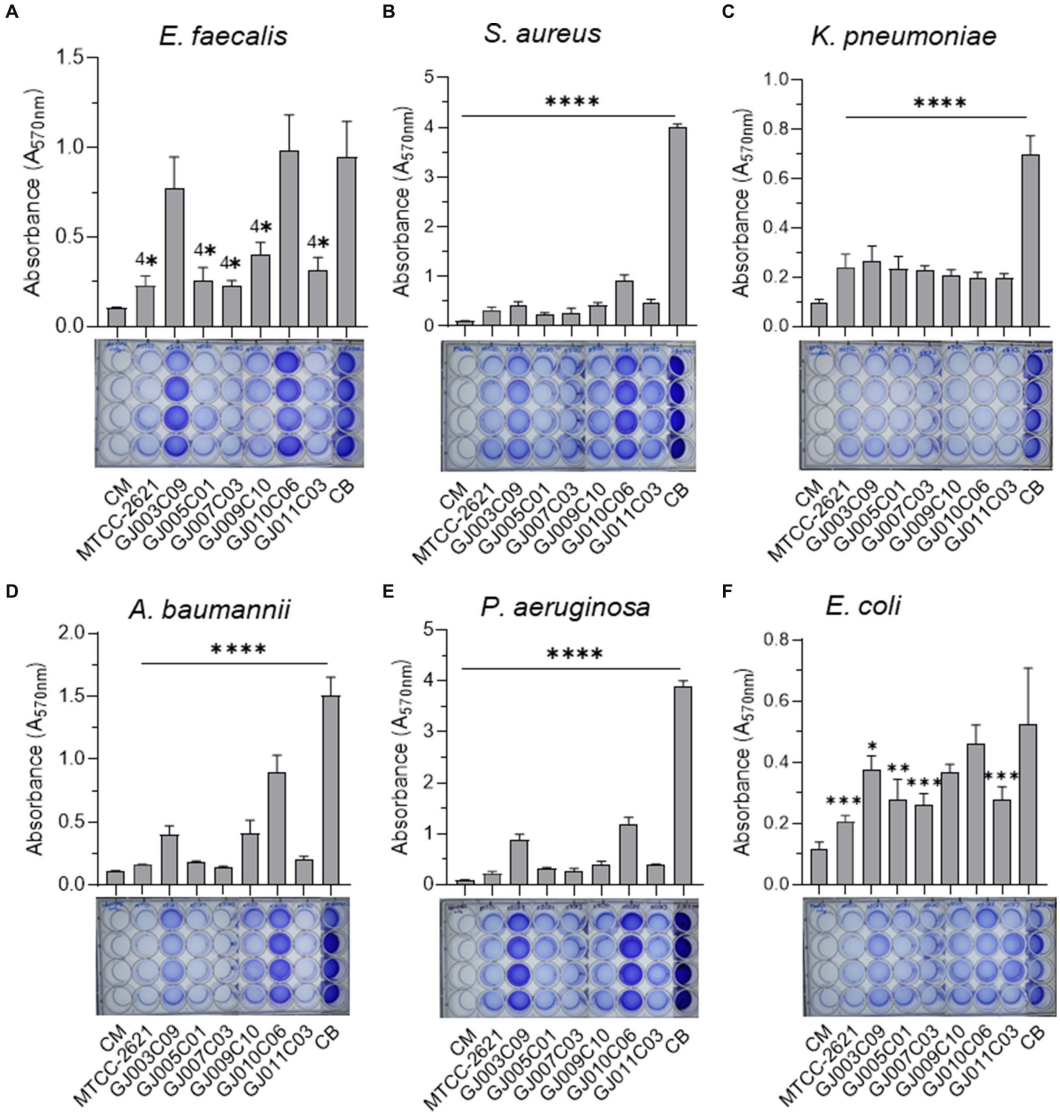
*Lactobacillus* isolates prevent biofilm formation by ESKAPE pathogens. The biofilm inhibition ability of *Lactobacillus* isolates was assessed using Cell-free culture filtrate (CSF). The assay was performed in 24-well polystyrene plates. *ESKAPE* pathogens [**(A)** E. faecalis, **(B)** S. aureus, **(C)** K. pneumoniae, **(D)** A. baumannii, **(E)** P. aeruginosa, and **(F)** E. coli] were cultured either in the presence or absence of Lactobacillus-CSF and incubated in static conditions at 37 ˚C for 48 hours. Subsequently, quantitative measurement of biofilm was performed using the standard crystal violet-based assay. The bar diagrams depict the Mean ± SD of the absorbance (A_570nm_) values from triplicate wells. The experiment was performed twice. One-way ANOVA was performed to compare the mean differences of each condition compared to the base value without any CSF (negative control biofilm, CB). *p < 0.05, **p < 0.01, ***p < 0.001, ****p < 0.0001. All *Lactobacillus* -CSF exhibited various degrees of biofilm inhibition on *ESKAPE* bacteria. *Lp plantarum (MTCC-2621)* used as a positive control.

*ESKAPE* pathogens are major causes of infection in animals and humans; however, the incidence of antibiotic failure against these pathogens is a critical public health problem (Manyi-Loh et al., 2018). These bacteria form biofilms, which make them resistant to antibiotics and immune cells. Probiotic *Lactobacillus* are known to inhibit the growth of pathogens via the production of antimicrobial substances such as organic acids, hydrogen peroxide, bacteriocins, and antimicrobial peptides (Plaza-Diaz et al., 2019). Antagonistic activity against such pathogens is a prerequisite for potential probiotics. Therefore, we screened the antagonistic activity against *ESKAPE* pathogen of the *Lactobacillus* isolates in two sets of experiments. First, the antagonistic growth potential of lactobacillus cell-free supernatant (CFS) and cell lysate was evaluated by an agar well diffusion assay (Supplementary Figure S5 and Figure 7), and second, anti-biofilm efficacy of CFS was evaluated by measuring their ability to prevent biofilm formation in a microplate-based assay (Figure 8). In the agar well diffusion assay, *Lactobacillus* isolates GJ009C10, GJ011C03, GJ005C01, and GJ007C03 demonstrated considerable levels of inhibition against the majority of *ESKAPE* pathogens in the case of both CFS (Figure 7A) and cell lysate (Figure 7B). In contrast, growth inhibition was relatively lesser for the isolates GJ003C09 and GJ010C06. Notably, CFS and cell lysate from all the *Lactobacillus* isolates exhibited highest inhibition of *A. baumannii*, among the *ESKAPE* pathogens. The zone inhibition data showed similar trends between CFS and cell lysate treatments (Figure 7).

Cell-free supernatants of *Lactobacillus* isolates exhibited anti-biofilm activity against *ESKAPE* pathogens (Figure 8). All *Lactobacillus* culture-free supernatants exhibited highly significant inhibition of biofilm formation by *E. faecalis*, *S. aureus*, *K. pneumoniae, A. baumannii,* and *P. aeruginosa,* although there was a certain degree of variation in their efficiency of inhibition (Figures 8A–E). In the case of *E. coli* biofilms, while the degree of inhibition was lower than that observed against the other five bacteria, the *Lactobacillus*—CFS of GJ007C03 and GJ011C03 showed considerable inhibition, followed by moderate biofilm inhibition observed with CFS of GJ003C01 and GJ009C10 (Figure 8F). Of the six *Lactobacillus* isolates, the CSF of four isolates, specifically GJ009C10, GJ011C03, GJ005C01, and GJ007C03, significantly (*p* < 0.01) reduced biofilm formation by *ESKAPE* pathogens.

## Discussion

The use of probiotics in animals has gained significant interest in recent years because of their numerous health benefits and market demands. While most commercial probiotics used in livestock are of human or dairy origin, probiotics from a similar host origin as the target host are preferred because of their greater evolutionary adaptability to the host’s gastrointestinal environment and superior biological activity (Dowarah et al., 2018). Probiotics isolated from animal intestines possess distinct characteristics, such as higher resistance to bile salts and low pH levels, as well as enhanced intestinal adherence abilities compared to dairy-based probiotics (Sornplang and Piyadeatsoontorn, 2016; Bazireh et al., 2020). Additionally, there is a growing need to develop species-specific probiotics capable of combating DR and MDR pathogens to improve the health and performance of livestock animals (Ripamonti et al., 2011; Blajman et al., 2015). Previous studies have reported the isolation and characterization of probiotic bacterial species from goat milk and feces (Setyawardani et al., 2011; Andrada et al., 2022); however, there are currently no reports on *Lactobacillus* species specifically derived from the jejunum intestinal compartment. Thus, this study aimed to isolate and characterize *Lactobacillus* species from goat small intestine jejunum, focusing on their morphological, biochemical, molecular, functional, and antagonistic properties against the proliferation and biofilm formation of the *ESKAPE* group of pathogens. The selection of probiotic bacteria was based on various criteria outlined by the Food and Agriculture Organization (FAO) and World Health Organization (WHO) (Bajagai et al., 2016). These criteria include bacterial origin, species, strain characterization, functional aspects (gastric acid and bile tolerance, carbohydrate utilization, and antimicrobial activity), antibiotic resistance, surface properties, and biosafety assessment (Bajagai et al., 2016). Here, out of 54 morphologically similar prospective *Lactobacillus* colonies that grew on MRS agar from the goat-jejunum tissue homogenates, following multi-layered and multi-parametric identification via molecular, microbiological, and biochemical methods, only six catalase negative isolates were shortlisted for further assessment of beneficial probiotic properties. Catalase-negative probiotics are considered superior as they can survive and thrive in the low-oxygen environment of the digestive system, and are also less likely to cause harmful changes in the gut microbiota by producing reactive oxygen species (ROS), which can damage the intestinal lining and trigger inflammation. These six isolates were gram-positive, bacillus, positive for esculin hydrolysis, and negative for indole production, nitrate utilization, and Voges Proskauer test. They could ferment complex carbohydrates and showed >99% sequence similarity with 16S rRNA gene and the 16S-23S ISR region for *Lactobacillus species.*

Probiotic candidates must tolerate low pH and bile salts to survive in the gastrointestinal tract (Chou and Weimer, 1999). Bacteria must survive the low pH of <2.0 in the stomach in the case of simple stomach animals, and < 2.5, in the abomasum in the case of ruminants, and remain viable for at least ~4 h before reaching the intestines. Furthermore, free bile acid that is synthesized in the liver conjugates with glycine or taurine, generating conjugated bile salts and releasing them in the duodenum (Urdaneta and Casadesús, 2017). These bile salts exhibit antimicrobial activity by damaging the bacterial cell walls and inducing DNA damage (Begley et al., 2005). To cope with bile salts, bacteria in the gut must possess an intrinsic resistance mechanisms (Ruiz et al., 2013). Certain probiotic strains, including *Lactobacillus* spp., have specific proteins devoted to the efflux of bile salts and protons, modifying sugar metabolism, and preventing the misfolding of proteins (Ruiz et al., 2013). In this study, the survival of the different *Lactobacillus* isolates varied under acidic and bile salt conditions. Some strains showed considerable survival at pH 2.0 and 3.0, while others demonstrated tolerance to different concentrations of bile salts, suggesting a strain-specific pattern. Notably, maximum survival or tolerance to acidity was observed in the case of GJ011C06 at a lower pH, and GJ009C10, GJ010C06, and GJ011C03 tolerated a higher bile salt environment than the other three *Lactobacillus* isolates.

To increase the chances of survival and colonization in the gastrointestinal tract, probiotic bacteria must adhere to the intestinal epithelium (Tuo et al., 2013). Both auto-aggregation and surface hydrophobicity testing serve as pre-tests for evaluating the adhesion capacity of probiotic bacteria to the epithelial cells. Various bacterial structures and components, such as pili, fimbriae, adhesins, mucus-binding proteins, fibronectin-binding proteins, surface layer proteins, lipoteichoic acid, and exopolysaccharides enable epithelial colonization (Krausova et al., 2019). This attachment serves a protective role by competing with intestinal pathways for host cell-binding sites. All the *Lactobacillus* isolates in our study exhibited hydrophobicity values ranging from 60 to 80% using both the Xylene and Hexane methods. Similar hydrophobicity values were reported in several earlier studies for *Lactobacillus* isolates (Gomaa, 2013), and *Lactobacillus* with >40% hydrophobicity were previously selected as supplement for swine feed (Dowarah et al., 2018). Furthermore, the *Lactobacillus* isolates exhibited significant auto-aggregation ranging from 60–80%, indicating their potential for colonization and adhesion in the gastrointestinal tract.

Adherence to epithelial cells and mucous has long been considered one of the selection criteria for probiotic microorganisms. It is an important requirement for both colonization and persistence inside gastrointestinal tract. Our findings showed that, *Lactobacillus* isolates GJ011C03 (74.4%) and GJ03C09 (73.3%) were strongly adhesive to MDBK cells, while moderate adhesion was observed for GJ009C10, GJ007C03 and GJ005C01, and least adhesion (45.5%) was observed for isolate GJ010C06. Varied levels of adhesion to mammalian cells were reported by several previous studies (Wang et al., 2009; Grover et al., 2011; Pringsulaka et al., 2015) Variations among adhesion can be seen between isolates as probiotic properties are generally strain specific within same species (Jacobsen et al., 1999). Although, the *in vitro* assays used for assessing the adherence potential of probiotic strains may not Truly mimic the gut environment, it provides valuable clue in short listing the potential probiotic strains for further validation in the animal models and livestock for their utility as direct-fed microbials.

Microbial communities, such as biofilms, when attached to a substratum or to each other, are effective in controlling biofilm formation by other bacterial species (Elbadri et al., 2019). *Lactobacillus* spp. are known to form robust biofilms compared to other bacteria (Kubota et al., 2008). Probiotic biofilms can enhance colonization, prolong persistence in the intestinal mucosa, and regulate pathogenic biofilms (Lagrafeuille et al., 2018). In this study, the *Lactobacillus* isolates exhibited varied levels of biofilm formation under both aerobic and anaerobic conditions; however, they had significantly higher levels of biofilm formation under anaerobic conditions, and the top three biofilm-forming *Lactobacillus* isolates were GJ005C01, GJ009C10, and GJ011C03. In addition to the anti-pathobiont effect and increased colonization due to enhanced biofilm formation by probiotic *Lactobacillus* in the gut under anaerobic conditions, it may exert other beneficial effects. These include (i) enhanced nutrient absorption: biofilms provide a protective layer and retain nutrients that may be lost in the conventional gastrointestinal tract, allowing better nutrient absorption by the probiotic bacteria; (ii) defense against antibiotics, which enables the probiotic bacteria to survive and flourish even in the presence of antibiotics, thereby maintaining their beneficial effects on the gut during antibiotic therapy; and (iii) biofilm-mediated communication: biofilms are known to facilitate communication among bacteria, allowing for better coordination of metabolic and physiological functions and enabling the probiotic bacteria to function more efficiently for better gut homeostasis (Barzegari et al., 2020).

*Lactobacillus* can acquire and spread antibiotic resistance genes through horizontal gene transfer (van Reenen and Dicks, 2011). Hence, antibiotic resistance testing is crucial when using new probiotic isolates as feed supplements, because of the potential risk of transferring antibiotic resistance to pathogenic bacteria and reducing the efficacy of antibiotics. According to EU regulatory frameworks, any bacterial strain carrying an acquired gene conferring AMR or strains with the unknown genetic nature of demonstrated resistance to antimicrobial agents should not be used as a feed additive because of the risk of horizontal spread (Stefańska et al., 2021). In this study, all caprine gut-probiotic *Lactobacillus* isolates were found to be susceptible to all the antibiotics listed in the EFSA panel, except vancomycin. Although vancomycin did not have breakpoints suggested by EFSA for *L. plantarum* obligate-, facultative-, heterofermentative, and homofermentative species, most of the *Lactobacillus* isolates showed intrinsic resistance to it [Tynkkynen et al., 1998; EFSA Panel on Additives and Products or Substances used in Animal Feed (FEEDAP), 2012]. Furthermore, screening for streptomycin MIC wasn’t essential for homofermentative *Lactobacillus* spp. [EFSA Panel on Additives and Products or Substances used in Animal Feed (FEEDAP), 2012], as an added caution, we evaluated the MIC for streptomycin, and were found to be below the cut-off values provided by EFSA. These findings on the selected *Lactobacillus* isolates, in addition to their non-hemolytic nature, indicate their potential use as feed supplements to promote animal health and prevent unwarranted emergence of antimicrobial resistance.

All six *ESKAPE* groups of pathogens commonly infect animals, with *Enterobacter* being the most common, followed by *K. pneumoniae, P. aeruginosa, S. aureus, E. faecium, and A. baumannii*. The major challenges of AMR in these pathogens are the multimodal antibiotic subversion mechanisms that have evolved, with biofilm formation being one of the leading causes. Therefore, agents that prevent biofilm formation and disperse preformed biofilms are associated with therapeutic benefits in animals. Components of the CFS of lactobacilli, such as exopolysaccharides and biosurfactants, may inhibit biofilm formation, as previously reported against MDR pathogens (Kaur et al., 2018; Rezaei et al., 2021). The Anti-biofilm effect of CFS of *L. rhamnosus and Lp. plantarum* is well-known against food pathogens, such as *P. aeruginosa* and *L. monocytogenes* (Rezaei et al., 2021). Probiotic *Lactobacillus* spp. have also shown inhibition of biofilm formation as well as a reduction in gene expression involved in the quorum sensing pathway in *Streptococcus mutans* (Wasfi et al., 2018). The Anti-biofilm effects of cell-free supernatants of *L. pentosus* and *Lp. plantarum* have also been reported against *B. cereus* and *P. aeruginosa* (Khiralla et al., 2015). In this study, the CFS of all six *Lactobacillus* isolates from goat small intestine inhibited biofilm formation by *ESKAPE* pathogens, with variation in inhibition ability among the strains. The present findings are in agreement with a previous report on *Lactobacillus* spp. isolated from the GI tract of other animal species (Dowarah et al., 2018).

In conclusion, this study has significantly advanced our understanding of the probiotic potential of *Lactobacillus* spp. isolated from the caprine gut, revealing not only their resilience under gastrointestinal-like stress but also their remarkable antimicrobial and biofilm inhibitory capacities. The standout isolates, GJ005C01, GJ007C03, GJ009C10, and GJ011C03, have shown exceptional promise, offering viable alternatives to traditional antibiotics in the livestock farming. These findings underscore the transformative potential of these probiotics in enhancing livestock health, with far-reaching implications for the food and pharmaceutical sectors. By harnessing the power of these probiotics, we can potentially mitigate the risks associated with emergence of antibiotic resistance, ushering in a new era of sustainable and effective disease management strategies in both agricultural and clinical settings.

## Data availability statement

The datasets presented in this study can be found in online repositories. The names of the repository/repositories and accession number(s) can be found in the article/Supplementary material.

## Ethics statement

All experiments were reviewed and approved by the Institutional Biological Safety Committee (IBSC, approval no. IBSC/2022/NIAB/BD/02) of the National Institute of Animal Biotechnology, Hyderabad. All procedures were performed in accordance with the relevant guidelines and regulations laid down by DBT-RCGM, Govt. of India. The requirement of the Institutional Animal Ethics Committee (IAEC) was waived because no animal experiments were involved, and only authorized slaughterhouse goat tissue samples were used for the isolation of probiotics.

## Author contributions

PS: Data curation, Formal analysis, Investigation, Methodology, Visualization, Writing – original draft, Writing – review & editing, Conceptualization. RA: Data curation, Investigation, Methodology, Writing – review & editing. RK: Data curation, Investigation, Methodology, Software, Writing – review & editing. SB: Formal analysis, Visualization, Writing – original draft, Writing – review & editing. VB: Data curation, Investigation, Methodology, Visualization, Writing – original draft, Writing – review & editing. BD: Conceptualization, Formal analysis, Funding acquisition, Methodology, Project administration, Resources, Software, Supervision, Writing – original draft, Writing – review & editing.
