# Supplementary material for: Restriction of growth and biofilm formation of ESKAPE pathogens by caprine gut-derived probiotic bacteria
Source: Front Microbiol. 2024 Jul 29;15:1428808. doi: 10.3389/fmicb.2024.1428808 (PMC11317286; doi:10.3389/fmicb.2024.1428808)
Supplement: Supplementary file 1 [file Data_Sheet_1.pdf]

Supplementary information

Restriction of the growth and biofilm formation of *ESKAPE* pathogens by caprine gut-derived probiotic bacteria

Authors:

Prerna Saini[1,2], Repally Ayyanna[1,†], Rishi Kumar[1,2], Sayan Kumar Bhowmick[1,2], Vinay Bhaskar[1], and Bappaditya Dey[1,2]*

[1]National Institute of Animal Biotechnology, Hyderabad, Telangana, India

[2]Regional Centre for Biotechnology, Faridabad, India

[†], Present Address: The University of Alabama in Huntsville, Chemical & Material Engineering, Huntsville, Alabama, USA

*Corresponding address:

Bappaditya Dey
Scientist-F, National Institute of Animal Biotechnology (NIAB)
Survey No. 37, Extended Q City Road, Gowlidoddi, Gachibowli
Hyderabad, Telangana, India 500032
E-mail: bdey@niab.org.in
Ph: +917042077704
ORCID ID # 0000-0003-2728-4683

Running title

Gut probiotics inhibit ESKAPE pathogens

Supplementary Table 1. PCR primers used for identification of *Lactobacillus* isolates

Name	Nucleotide Sequence	Target	Reference
***Lactobacillus* genus specific PCR primer**			
**R16-1**	CTTGTACACACCGCCCGTCA	16s-rRNA gene	Dubernet et al., 2022
**LbLMA1-rev**	CTCAAAACTAAACAAAGTTTC		
***Lactobacillus sp.* PCR and sequencing primer**			
**16S(ISR)-F**	GCTGGATCACCTCCTTTC	16S-23S ISR	Gurtler et al., 1996<br>Turner et al., 1999<br>Weisburg et al., 1991
**23S(ISR)-R**	CCTTTCCCTCACGGTACTG		
**16S(8-27)-F**	AGAGTTTGATCCTGGCTCAG	16-rRNA-V1-V3 region	
**V3(519-536)-R**	GTATTACCGCGGCTGCTG		

Supplementary Fig. 1

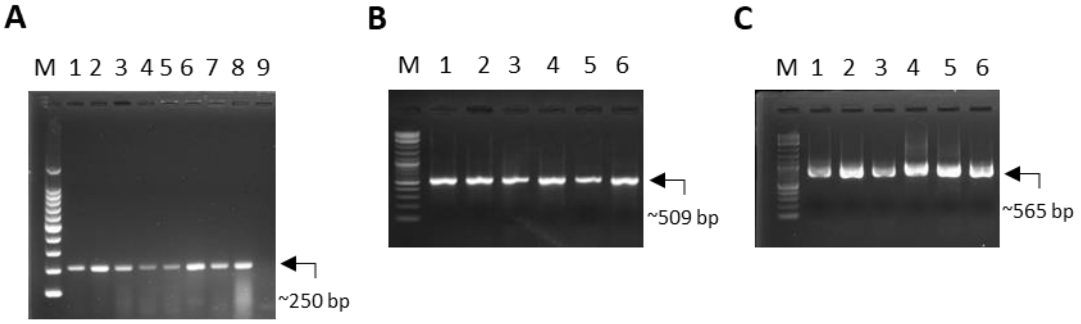

Supplementary Fig. 1. PCR and agarose gel electrophoresis analysis for molecular identification of LAB isolates. Representative agarose gel electrophoresis analysis images of (**a**) *Lactobacillus* genus-specific PCR analysis using 16S-rRNA gene-specific primers, (**b**) 16S rRNA gene V1-V3 region, and (**c**) 16S-23S ISR of genomic DNA from LAB isolates. Following is the lane description.

A. 1. *Lp. plantarum MTCC-2621* (+ve control), 2. *GJ003C09,* 3. GJ005C01, 4. GJ007C03, 5. GJ009C10, 6. GJ010C06, 7. GJ011C03, 8. GJ010C02, 9. PCR –ve control.
B. 1. *GJ003C09,* 2. GJ005C01, 3. GJ007C03, 4. GJ009C10, 5. GJ010C06, and 6. GJ011C03
C. 1. *GJ003C09,* 2. GJ005C01, 3. GJ007C03, 4. GJ009C10, 5. GJ010C06, and 6. GJ011C03

Supplementary Fig. 2

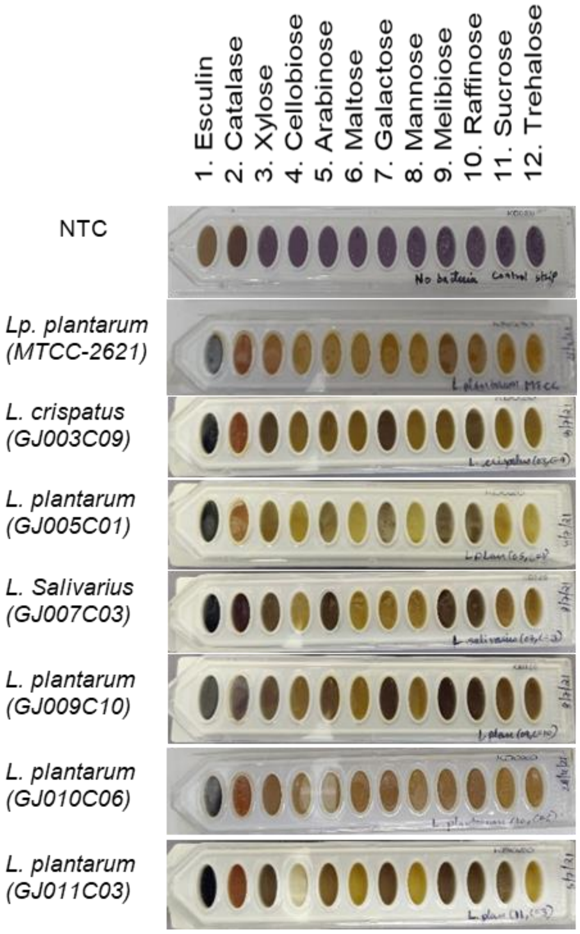

Supplementary Fig. 2. Carbohydrate fermentation ability of *Lactobacillus* isolates. The carbohydrate fermentation ability of Lactobacillus isolates was assessed by using the Hilacto identification kit (KB020, Himedia). One assay strip has 12 wells including 10 different carbohydrate sugars, one for esculin, and the other one for catalase. The carbohydrate fermentation was measured via colorimetric identification based on the principle of pH change and substrate utilization for the genus Lactobacillus. While dark coloration confirms esculin hydrolysis (Lane -1), light coloration indicates sugar fermentation (Lane 3-12). For the catalase test visual observation of effervescence was considered a positive test, and no effervescence for the negative catalase test (Lane- 2). All 6 *Lactobacillus* isolates were found positive for esculin, negative for catalase, and with varied abilities to ferment different types of sugar. NTC: Negative control, and *Lp. plantarum* (*MTCC-2621*): positive control.

Supplementary Fig. 3

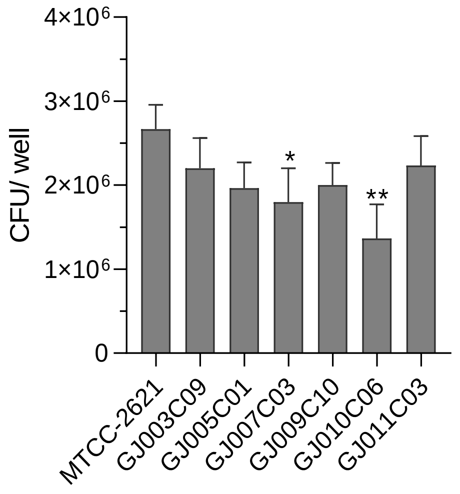

Supplementary Fig. 3. CFU of epithelial cell adhered *Lactobacillus* isolates. MDBK cells were incubated with fluorescently labeled probiotic isolates at an MOI of 1:10 for 2 hours and subsequently after thorough washing, adhered cell numbers were measured via CFU plating. The bar diagram depicts the mean ± SD of total CFU / well in triplicates. ANOVA test was performed to compare the mean of *Lactobacillus* isolates to that of MTCC-2621 positive control strain. *, $p < 0.05$; **, $p < 0.01$.

Supplementary Fig. 4

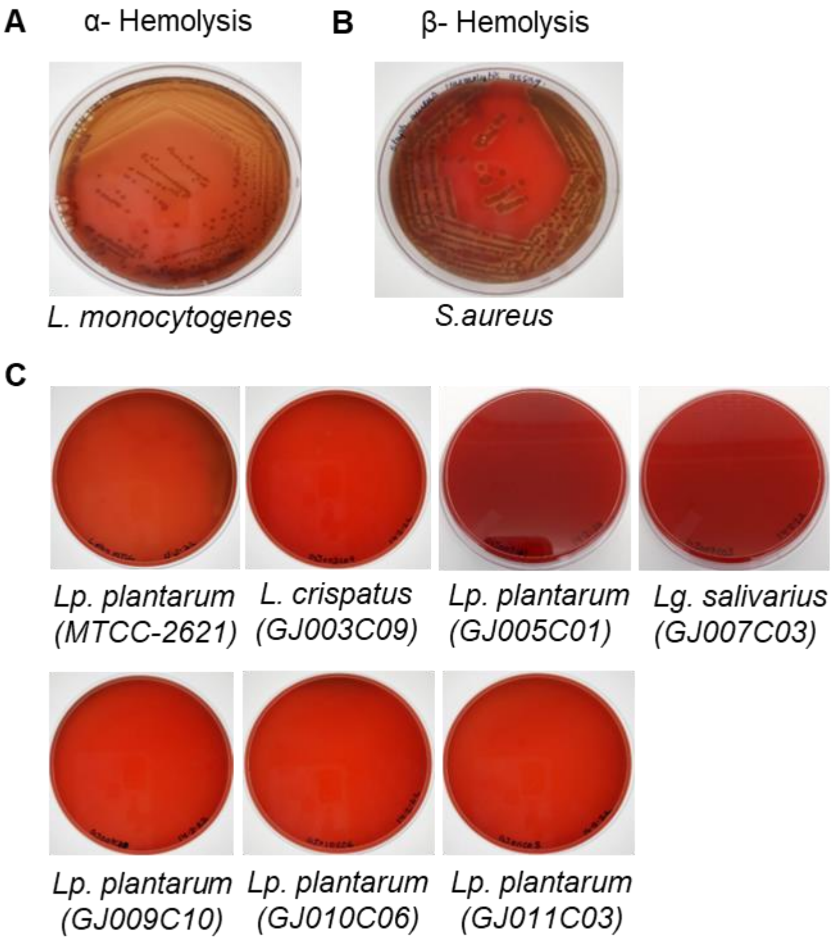

Supplementary Fig. 4. Caprine gut-derived *Lactobacillus* isolates are non-hemolytic. Overnight-grown *Lactobacillus* cultures were streaked onto blood agar media containing 5% goat blood and incubated at 37˚C for 24 hours. Two positive control bacteria, (A) *Listeria monocytogenes,* and (B) *Staphylococcus aureus*, were used for α- and β- hemolysis, respectively. The clear and colored zones surrounding the colonies were examined. Clear zones were indicative of beta hemolysis, greenish zones were indicative of alpha hemolysis and the absence of zones indicated no hemolysis or gamma hemolysis. (C) All 6 Lactobacillus isolates including the *Lp. plantarum (MTCC-2621)* positive control did not show any hemolysis on the blood-agar plates.

Supplementary Fig. 5

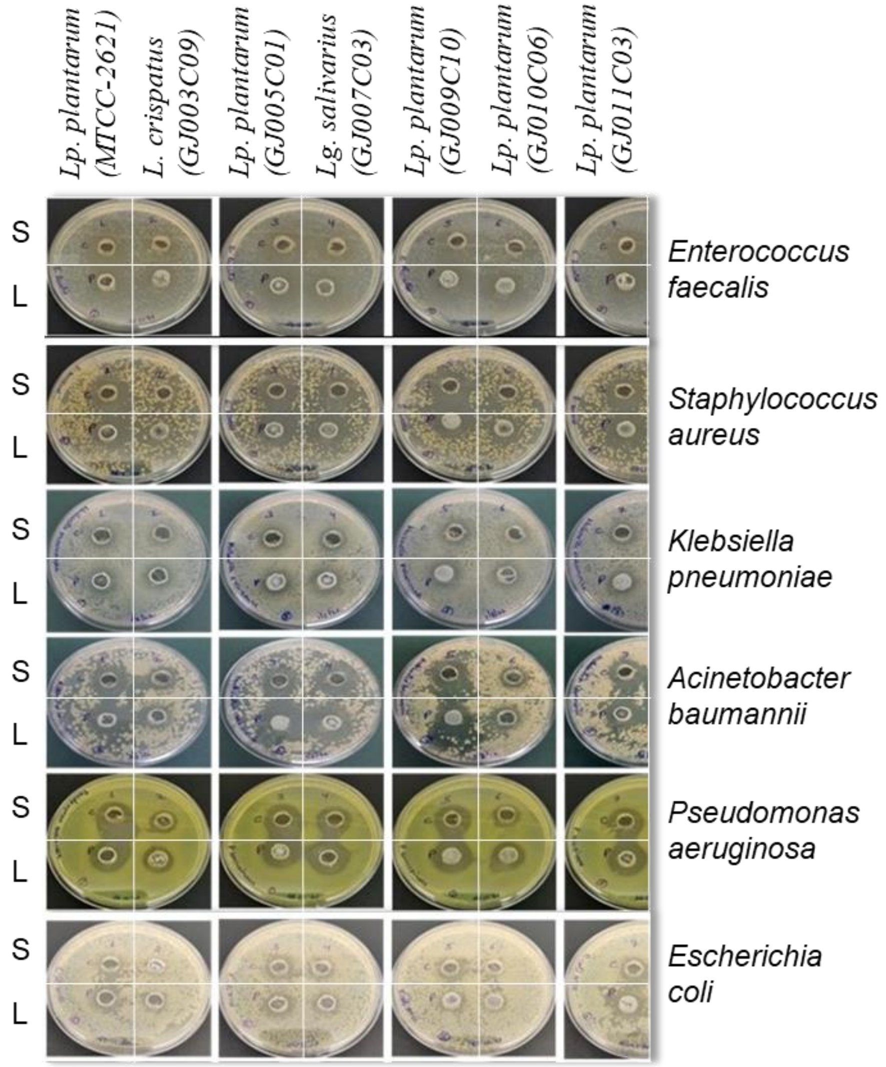

Supplementary Fig. 5. *Lactobacillus* isolates restrict the growth of *ESKAPE* pathogens. The figure depicts representative images of nutrient agar plates from agar well diffusion assay-based antimicrobial activity assay. Each *ESKAPE* bacterial inoculum was spread onto nutrient agar plates. Subsequently, 10 mm diameter wells were made on the agar plates using 1 ml sterile pipette tip bottoms. Sterile-filtered cell-free culture supernatants (S) and cell lysate (L) of *Lactobacillus* isolates were added to each well and incubated for 24 hours at 37˚C in aerobic conditions. The zone of inhibition was recorded with the zone of inhibition scale. *Lp. plantarum (MTCC-2621)* was used as a reference strain. Experiments are performed thrice.

References

Dubernet, S., Desmasures, N., and Guéguen, M. (2002). A PCR-based method for identification of lactobacilli at the genus level. *FEMS Microbiol. Lett*. 214, 271–275. doi: 10.1111/j.1574-6968.2002.tb11358.x

Gurtler, V., and Stanisich, V. A. (1996). New approaches to typing and identification of bacteria using the 16S-23S rDNA spacer region. *Microbiology (Reading)* 142, 3–16. doi: 10.1099/13500872-142-1-3

Turner, S., Pryer, K. M., Miao, V. P. W., and Palmer, J. D. (1999). Investigating deep phylogenetic relationships among cyanobacteria and plastids by small subunit rRNA sequence analysis. *J. Eukaryot. Microbiol*. 46, 327–338. doi: 10.1111/j.1550-7408.1999.tb04612.x

Weisburg, W. G., Barns, S. M., Pelletier, D. A., and Lane, D. J. (1991). 16S ribosomal DNA amplification for phylogenetic study. *J. Bacteriol*. 173, 697–703. doi: 10.1128/jb.173.2.697-703.1991
